# Coexistence of chronic hyperalgesia and multilevel neuroinflammatory responses after experimental SCI: a systematic approach to profiling neuropathic pain

**DOI:** 10.1186/s12974-022-02628-2

**Published:** 2022-10-29

**Authors:** Lei Wang, Mehmet A. Gunduz, Ana T. Semeano, Enis C. Yılmaz, Feras A. H. Alanazi, Ozan B. Imir, Ulas Yener, Christian A. Arbelaez, Esteban Usuga, Yang D. Teng

**Affiliations:** 1 Department of Physical Medicine and Rehabilitation, Harvard Medical School, Boston, MA USA; 2 Laboratory of SCI, Stem Cell and Recovery Neurobiology Research, Department of Physical Medicine and Rehabilitation, Spaulding Rehabilitation Hospital Network, Mass General Brigham, and Harvard Medical School, 300 1St Avenue, Charlestown Navy Yard, Boston, MA 02129 USA; 3 Neurotrauma Recovery Research, Department of Physical Medicine and Rehabilitation, Spaulding Rehabilitation Hospital Network, Mass General Brigham, and Harvard Medical School, Boston, MA USA

**Keywords:** Spinal cord injury, Neurogenesis, Neuroinflammation, Neuropathic pain, Neuroplasticity, Rat

## Abstract

**Background:**

People with spinal cord injury (SCI) frequently develop neuropathic pain (NP) that worsens disability and diminishes rehabilitation efficacy. Chronic NP is presently incurable due to poor understanding of underlying mechanisms. We hypothesized that multilocus neuroinflammation (NIF) might be a driver of SCI NP, and tested it by investigating whether NP coexisted with central NIF, neurotransmission (NTM), neuromodulation (NML) and neuroplasticity (NPL) changes post-SCI.

**Methods:**

Female Sprague–Dawley rats (230–250 g) with T10 compression or laminectomy were evaluated for physical conditions, coordinated hindlimb functions, neurological reflexes, and mechanical/thermal sensitivity thresholds at 1 day post-injury (p.i.) and weekly thereafter. Eight weeks p.i., central nervous system tissues were histochemically and immunohistochemically characterized for parameters/markers of histopathology and NIF/NTM/NML/NPL. Also analyzed was the correlative relationship between levels of selected biomarkers and thermosensitivity thresholds via statistical linear regression.

**Results:**

SCI impaired sensorimotor functions, altered reflexes, and produced spontaneous pain signs and hypersensitivity to evoked nociceptive, mechanical, and thermal inputs. Only injured spinal cords exhibited neural lesion, microglia/astrocyte activation, and abnormal expression of proinflammatory cytokines, as well as NIF/NTM/NML/NPL markers. Brains of SCI animals displayed similar pathophysiological signs in the gracile and parabrachial nuclei (GrN and PBN: sensory relay), raphe magnus nucleus and periaqueduct gray (RMN and PAG: pain modulation), basolateral amygdala (BLA: emotional-affective dimension of pain), and hippocampus (HPC: memory/mood/neurogenesis). SCI augmented sensory NTM/NPL (GrN and PBN); increased GAD67 (PAG) level; reduced serotonin (RMN) and fear-off neuronal NTR2 (BLA) expressions; and perturbed neurogenesis (HPC).

**Conclusion:**

T10 compression caused chronic hyperalgesia that coexisted with NIF/NTM/NML/NPL responses at multilevel neuroaxis centers. The data have provided multidimensional biomarkers as new mechanistic leads to profile SCI NP for therapeutic/therapy development.

**Supplementary Information:**

The online version contains supplementary material available at 10.1186/s12974-022-02628-2.

## Introduction

Traumatic spinal cord injury (SCI), either moderate or severe, can produce long-term sensorimotor deficit, autonomic abnormality, respiratory disorder, lower urinary tract dysfunction, and additional debilitating sequelae including neuropathic pain (NP) and mood disorder [1, 2]. It has been reported that ≥ 80% of all individuals surviving acute SCI experience severe pain, and 40–60% of them develop chronic pain [3]. Notably, chronic NP, which remains incurable, has been diagnosed in ≥ 53% of people with SCI, jeopardizing life quality and rehabilitation opportunity, and imposing socioeconomic burden [1–3]. Effective clinical management for SCI NP and control of its biological and/or psychological triggers are unmet medical needs [4–6]. In therapeutic development, laboratory interventions have aimed to suppress specific neural facilitation of pain [7], activate inhibitory interneurons and neuromodulation (NML) [8, 9], reduce pain neurotransmission (NTM) [10, 11], or impede neuroinflammation (NIF) [12]. Therefore, the broad mechanistic diversity of post-injury (p.i.) NP has likely played a major role in preventing conventional therapeutic tactics from exerting long-term efficacy and avoiding serious side effects [13–15].

An earlier study of ours demonstrated that administration of the multimodal effecting drug huperzine A, which concurrently stimulates inhibitory interneurons and suppresses NIF via elevating cholinergic tone, and mitigates excitotoxicity and sensory neuronal activity by blocking NMDA receptors, ameliorated NP in rats with T10 compression [14]. Clinically, neuroimaging uncovered that SCI NP increased intraspinal cord NIF metabolite levels [15]. These data suggested that local and systemic inflammation may act as pathogenic factors to induce the evolvement of NP, pain-triggered complications, and other neural disorders p.i. (e.g., affective components of pain, distal motor neuron/neuromuscular junction abnormalities, and axon demyelination) [15–22]. However, to date, little has been done to systematically investigate NIF in the central nervous system (CNS) using clinically relevant SCI models. Here, we hypothesized that NIF in central sensory relay and pain modulation nuclei might be a primary driver of chronic SCI NP, and tested the hypothesis by first probing whether T10 compression caused long-term coexistence of NP and multilocus NIF responses in female rats. Since human NP is difficult to model [23], we utilized physical signs of pain, evoked hypersensitivities, plus sensorimotor and NIF/NTM/NML/NPL outcome measures to generate multidimensional biomarkers as novel mechanistic leads to systematically profile SCI NP, aiming to improve the study’s clinical relevance [24].

## Methods and materials

### Animals, SCI modeling, and perioperative managements

Young adult female Sprague–Dawley (SD) rats (230–250 g; *n* = 24) were purchased from Charles River Laboratories (Wilmington, MA), and housed in pairs at the Animal Research Center of the Boston Children’s Hospital under a 12-h light/dark photocycle in a colony under ambient temperature and humidity with food and water available ad libitum [14, 25, 26]. T10 compression was performed according to a standardized protocol [14, 26]. Concisely, rats were anesthetized with ketamine (75 mg/kg, i.p.)/xylazine (10 mg/kg, i.p.) and placed on a heating pad (37 °C) that was irrigated with an EZ-200 water circulation pump (E-Z Systems Inc., Palmer, PA, USA). Buprenorphine was administered (0.05 mg/kg, s.c.) for pre-emptive analgesia. A midline incision, soft tissue dissection, and laminectomy were made at the 10th thoracic vertebral level (T10: corresponding to T11 spinal cord). Hemostasis was induced with normal saline and Gelfoam^®^ (Pfizer). After the body trunk was suspended (~ 1 cm clearance) with a Rodent Spine Stabilization Frame custom-made by the Scientific Instrument Shop, Harvard University School of Engineering and Applied Sciences (Cambridge, MA) [26], a 35 g stainless steel impounder was lowered gently upon the dura via a micromanipulator (MM 33, Märzhäuser) to deliver a 5-min standing load of the weight (i.e., a moderate compression regimen) [26]. For rats that received T10 laminectomy only as sham surgery controls, no weight loading was performed. The muscles were then sutured (3-0 Ethilon black brand, Ethicon^®^), and skin stapled with wound clips (Reflex^®^; Cellpoint Scientific, Gaithersburg, MD) [14, 26].

All animals were returned to a clean, heated cage with readily accessible drinking water and hydrated soft food (DietGel® Recovery, ClearH_2_O Inc., Westbrook, ME) for postoperative recovery. Lactated Ringer’s solution (10 mL/rat, s.c.; Abbott Laboratories, Chicago, IL) and buprenorphine HCl (0.05–0.10 mg/kg, s.c., q12h.) or buprenorphine slow-release (1.2 mg/kg, s.c., q70–72 h) were given for 5 days. The bladder was evacuated manually without catheterization (b.i.d.) until establishment of an emptying reflex, for which shorter urethral length in female rats (versus males) enables more efficient recovery [14, 26, 27]. No prophylactic antibiotics were used.

Following the animal protocol, signs of stress and pain, which included reduced grooming, porphyrin staining around the eyes, vocalization, etc., were routinely monitored and analyzed based on a formula we developed to determine the likelihood of spontaneous pain presence (Additional file 1: Table S1).

### Experimental design

**Fig. 1:**
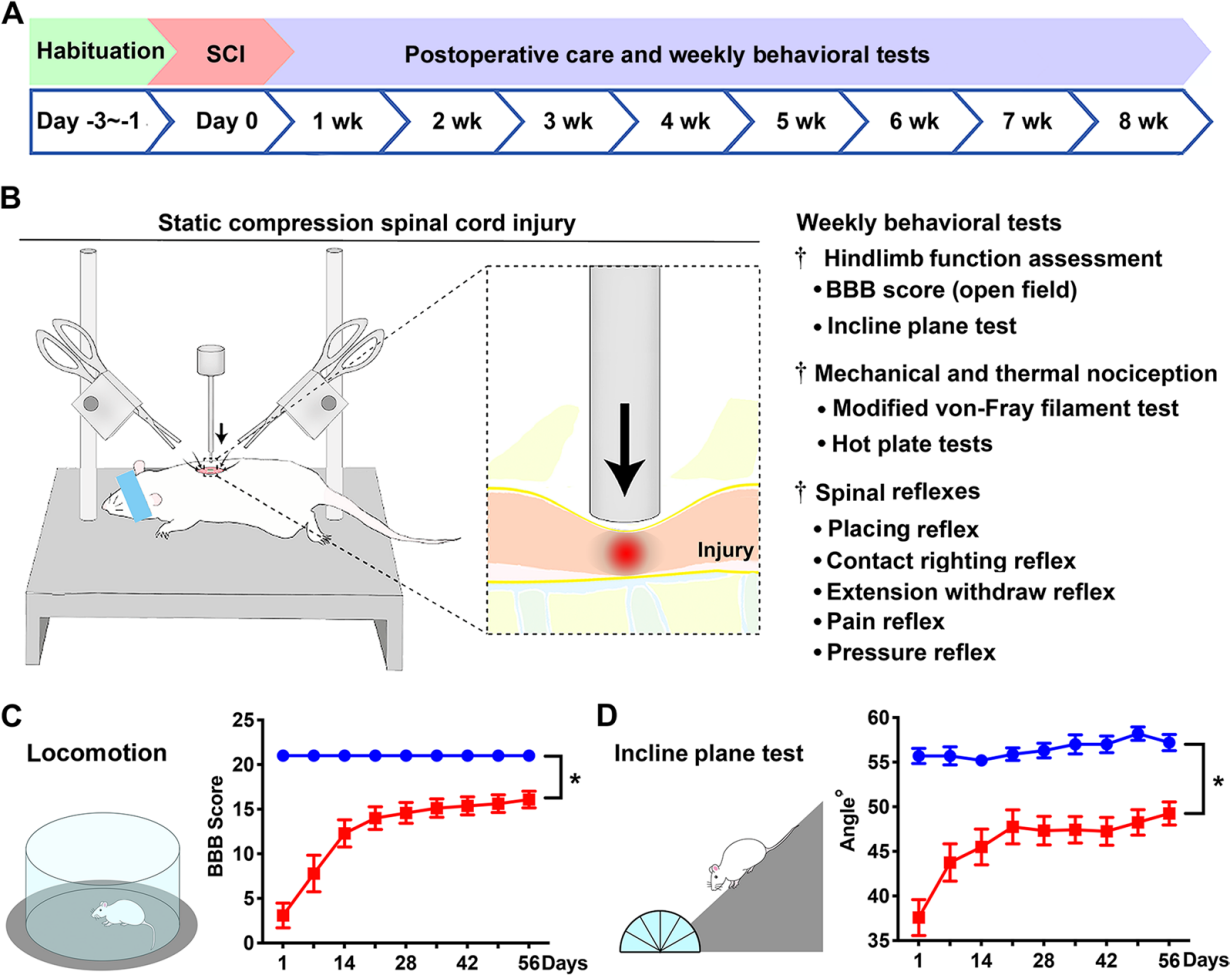
The experimental design, SCI modeling, and coordinated hindlimb functional changes. **A** The time course of major experimental procedures comprised a brief habituation, T10 compression and postsurgical care, and behavioral evaluations performed weekly for 8 weeks. **B** Left: this schematic diagram illustrated the surgery setting of T10 moderate quasi-static compression (35 g × 5 min). Middle: a zoomed in presentation of the compression injury epicenter. Right: the contents of the performed behavioral tests. **C** The day 1 and weekly post-injury (p.i.) group average BBB locomotor scores of the SCI rats (red) versus those of the laminectomy control animals (blue) showed significant locomotion deficits resulting from SCI. **D** Left: the inclined plane test was done with rats facing downward. Right: compared to the laminectomy sham surgery, T10 compression resulted in significantly reduced group mean maximum angle where the animals could hold their position steady for 5 s (for both BBB and incline plan tests: *p < 0.05 to < 0.001; two-way repeated measures ANOVA with Sidak’s post hoc test; *n* = 12/SCI group; *n* = 10/laminectomy control group)

The experiments were conducted largely following a randomized block design. The sample size was determined on the basis of a power analysis from a previous study on the same SCI model [14]. With the α value (probability of rejecting *H*_0_ when *H*_0_ is true) being set at 5%, an average sample value of 0.41 g (post-SCI baseline sensory threshold), mean test value (post-treatment) of 3.1 g, and group size of four rats, the statistical power (i.e., 1-β) equaled ∼ 100%, indicating that the β value (i.e., the probability of not rejecting *H*_0_ when *H*_0_ is false) equaled ∼ 0%. All animals were habituated for ≥ 3 days, during which the baseline data were collected, which included body weight and outcomes of modified von Frey filament (mVF), locomotion, inclined plane, spinal reflex, and hot plate tests (Fig. 1A). All qualified rats received 3 rounds of functional magnetic resonance imaging [fMRI] examinations (pre-surgery, and 2 and 8 weeks after operation) for a separate study conducted by investigators of an independent laboratory.

The SCI and laminectomy control groups (*n* = 14 and 10, respectively) were behaviorally tested at 1 day and weekly thereafter for 8 weeks p.i. (Fig. 1A, B). Behavioral evaluation was performed during the 12-h light photocycle, and mostly by two examiners to obtain mutually agreed scores following the same test order (i.e., mVF → open-field locomotion → incline plate → proprioceptive positioning/placing reflex → other neurological reflexes →  ~ 30-min break → bilateral hot plate →  ~ 30-min break → unilateral hot plate; note: per convention, motor and sensory data were separately presented). At the end of the study (≥ day 56 p.i.), animals were euthanatized, and the spinal cord and brain tissues were fixed by intracardiac perfusion. All animals except two were investigated over the entire study duration (*n* = 22: 12/SCI and 10/laminectomy). For the two animals excluded, one was inadequately injured (i.e., no loss of micturition reflex), and the other was euthanatized due to a skin lesion (note: its data were not included). Data values were expressed as mean ± SEM. Statistical tests were conducted with significance level set at *p* < 0.05. The experimental procedures were performed in strict accordance with the *Guide for the Care and Use of Laboratory Animals* (US National Research Council Committee for the Update of the Guide for the Care and Use of Laboratory Animals, 8th edition) after review and approval by the Institutional Animal Care and Use Committee (IACUC) of Boston Children’s Hospital and the US Department of Defense.

### Evaluation of coordinated hindlimb functions

#### Locomotion

The hindlimb locomotion was assessed with the Basso, Beattie and Bresnahan (BBB) locomotor rating scale following the original protocol [28]. Briefly, animals were individually placed in an open, flat area staged by a rubber pad with a patterned surface texture (Fig. 1C). Bilateral hindlimb locomotor function was scored according to a 21-point scale (i.e., 0 for complete paralysis and 21 indicating normal function). The score comprehensively demonstrated hindlimb open-field locomotor aptitude.

#### Incline plane test

This assay quantifies a rat’s ability to maintain coordinated body posture control on an inclined plane (Fig. 1D) [25–27]. Specifically, animals were individually positioned facing downward on a 40 × 30 cm plastic board covered with a rubber mat that had a fine groove surface pattern. The steepest degree of inclination at which a rat could hold its body posture for 5 s on two consecutive trials when placed vertically on the board and facing downward was recorded [25–27].

### Measurement of sensory functions

#### Neurological reflexes

**Fig. 2:**
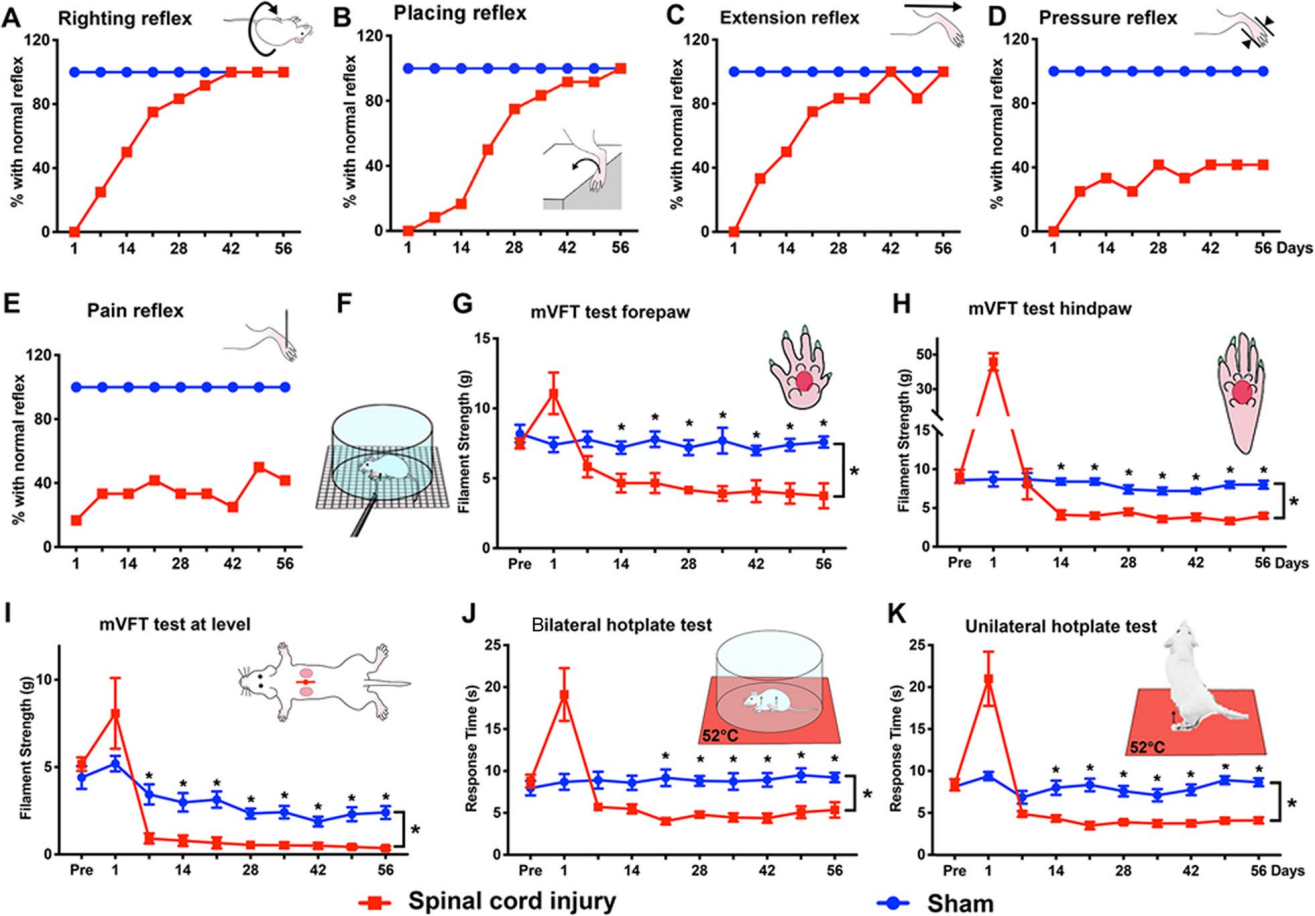
The data of sensorimotor behavioral evaluations of neurological reflexes, mechanical hypersensitivity, and bilateral or unilateral thermal hypersensitivity. **A**–**E** Spinal neurological reflexes. At day 1 post-injury (p.i.), SCI rats showed severe loss of contact righting reflex (**A**), proprioceptive positioning reflex of paw placing (**B**), and withdrawal reflex in response to brief extension of joints (**C**), which recovered overtime, with 80% SCI rats performing normally by 3 (**A**, **C**) or 4 weeks p.i. (**B**). Withdrawal reflexes caused by pressuring (**D**) and brief pinching (**E**) of the hindpaw exhibited discernible abnormalities p.i., with their recovery rates noticeably slower and more incomplete, relative to that of the righting (**A**), placing (**B**), or extension (**C**) reflex. By 8 weeks p.i., only ~ 33% SCI rats showed a normal nociception reflex (**E**). **F**–**H** Results of the modified von Frey filament tests of mechanical hypersensitivity (mVFT; **F**). In contrast to a stable array of sensitivity thresholds of the control group, at day 1 p.i., the mean threshold to trigger a withdrawal response following mVF stimulation drastically increased in the forepaws (**G**; 11.1 ± 1.5 g) and hindpaws (**H**; 45.8 ± 5.1 g) in the SCI rats. Afterward, the group mean threshold, compared to the pre-SCI level and control group, significantly decreased in the forepaws (i.e., above-injury level allodynia; **G**) and hindpaws (i.e., below-injury level allodynia; **H**), starting in 2 weeks after SCI. At-injury level allodynia measured in the dorsal dermatomes adjacent to the T10 injury had a comparable pattern of hypersensitivity (**I**; *p < 0.01 to < 0.001; *n* = 12/SCI or 10/control; two-way repeated measures ANOVA with Sidak’s post hoc test). **J**, **K** Data of bilateral and unilateral hot plate tests (i.e., thermal hypersensitivity/allodynia). In the bilateral hot plate test, the pre-SCI and pre-laminectomy mean hindlimb latencies were statistically indistinguishable and the control group showed steady postsurgical response latencies (**J**). At day 1 p.i., the group mean latency markedly increased (19.1 ± 3.1 s) in the injured rats. Starting in week 3 after SCI, thermal hypersensitivity developed, which remained continuous and significantly different than the baseline values and those of the control group (**J**; SCI: *n* = 12, laminectomy: *n* = 10; *p < 0.05–0.001; two-way repeated measures ANOVA with Sidak’s post hoc test). The unilateral hindlimb hot plate test revealed a similar pattern of response latency changes. However, this test detected significantly reduced group mean latencies 1 week earlier, starting on day 14 p.i., compared to the baseline level and control group (**K**; *n* = 12/SCI or 10/control; *p < 0.05 to < 0.001; two-way repeated measures ANOVA with Sidak’s post hoc test; note: to make the figure succinct, the gentle body holding logistics were not depicted in the inset)

The reflexes were measured per previously published formulas [14, 25–27]. The tests included postural reflex of contact righting (Fig. 2A), proprioceptive positioning reflex of paw placing, and hindlimb withdrawal reflexes to brief stimulations of extension, pressure, and pinch/nociception (Fig. 2B–E). All reflexes were evaluated as per our established scoring system (i.e., 0: areflexia; 1: hyporeflexia; 2: normoreflexia; or 3: hyperreflexia, among which the score of 2 was considered normal and scores of 0, 1, and 3 abnormal) [25–27].

#### mVF test (mVFT) of mechanical hyperalgesia and tactile allodynia

Rats were placed on a metal mesh floor inside a Plexiglas box. After ~ 3 min of acclimation, a series of von Frey hairs, which are nylon monofilaments of 50 mm in length and in varied diameters/stiffnesses (i.e., logarithmically incremental stiffness scaling from 0.008 to 300 g) exerting different forces (typical force range for the presented study: 0.04–60 g), were applied to the plantar surface of the forepaw and hindpaw, and dorsal skin of the T11–T12 dermatomes to detect above-, below-, and at-injury level hypersensitivity, respectively (Fig. 2F–I) [14]. The filament was applied with sufficient force to cause the fiber to bend to produce a punctate stimulus to a given area of the body for a total of ~ 5 s. Recorded as positive reactions were brisk withdrawal, paw licking, or body flinching that were evoked by the minimum threshold (unit: g).

#### Hot plate test to measure thermal hypersensitivity

The hot plate test utilizes latency measurements to evaluate acute, cutaneous hypersensitivity involving the supraspinal neural network response in rodents to noxious thermal stimuli [29]. In this study, bilateral and unilateral hot plate tests were done following published protocols [30, 31]. The metal plate surface of the apparatus (IITC Life Science, Woodland Hills, CA) was maintained at 52 °C. In the bilateral test (Fig. 2J), rats were individually habituated on a non-heated (i.e., room temperature) metal surface for 5 min before each was placed on the pre-heated plate with its locomotion territory restricted by a Plexiglas arena. The temporal latency to hindpaw licking and quick shaking, or jumping, was recorded, at which point the rat was swiftly removed. In the unilateral test (Fig. 2K), each rat was gently restrained to allow the plantar side of the tested hindpaw to contact the hot plate (52 °C) to measure the withdrawal latency of each paw separately, with the shorter one recorded as the animal’s thermosensitivity threshold. Three tests for either side were conducted separately and alternately at 2-min intervals in order to obtain the mean latency time as results. To prevent any occurrence of excessive thermal pain or injuries, the maximum time for each round of heat exposure was restricted to 30 s for both test types [30, 31].

### Histopathology

#### Tissue preparation

After all behavioral tests were completed 8 weeks p.i., animals were euthanatized with i.p. injection of 90 mg/kg ketamine and 15 mg/kg xylazine. Tissue was fixed via intracardiac perfusion with phosphate buffer (PB; pH 7.4) and 4% paraformaldehyde (PFA; pH 7.4). Afterward, spinal cords and brains were collected for overnight post-fixation in 4% PFA, followed by sequential dehydrations in 10–30% sucrose solutions before freezing in 2-methylbutane/isopentane (Sigma-Aldrich, St. Louis, MO) at − 50 °C for cryostorage (− 80 °C). For microscopic morphometric and immunohistochemical (IHC) analyses, the tissues were embedded in Tissue-Tek OCT compound (Sakura Finetek USA, Torrance, CA). Spinal cord cryosectioning generated circulation series of 20-μm tissue slices of paired injured and laminectomy spinal cords (5 section pairs/per slide × 10; frosted glass slides; Thomas Scientific, Swedesboro, NJ). The arrangement produced sections that comprised ∼ 1000 μm thickness of the spinal cord in each series of 10 slides [25–27]. This formula was adapted for brain sectioning: each series consisted of 5 slides with each having 3 × 40-μm-thick sections (i.e., 25 sections per series of ∼ 1000 μm thickness of brain tissue; tissue collection rate: 60%).

#### Histopathological analysis

For morphological analysis, every 10th (spinal cord) and 5th (brain) tissue section slides of each animal were stained with solvent blue (Sigma-Aldrich, St. Louis, MO) plus hematoxylin and eosin (H&E; Sigma-Aldrich, St. Louis, MO) as per our standard protocols [26, 32]. The stained tissue sections were imaged on an Axiovert 200 microscope with a digital Axiocam camera (Carl-Zeiss Microimaging). Image analysis was conducted using ImageJ^®^ (National Institutes of Health) and Photoshop^®^ (Adobe, San Jose, CA) to identify the lesion epicenter and quantify gray matter and white matter sparing as per methods previously published [27].

#### IHC assays

To detect markers of interest, standardized IHC assays were performed on tissue sections sampled from spinal cord loci within 1 mm range at 5 mm rostral to the lesion epicenter [14, 25, 26]. For analysis of the brainstem, midbrain, and cerebrum tissues, coronal sections were selected according to standard coordinates provided by “Rat Brain Atlas—gaidi.ca” (http://labs.gaidi.ca/rat-brain-atlas/; source: George Paxinos and Charles Watson. The Rat Brain in Stereotaxic Coordinates. Elsevier, 2006) with confirmation via microscopic verification to ensure inclusion of the gracile nucleus (GrN), parabrachial nucleus (PBN), raphe magnus nucleus (RMN or RMg), periaqueduct gray (PAG), hippocampus (HPC), or basolateral amygdala (BLA).

Per established protocols [14, 22], the paired sections were quickly washed, hydrated, and perforated using phosphate-buffered saline (PBS) with 0.03% Triton X-100 (PBST; Sigma-Aldrich) before blocking non-specific binding sites with 4% (vol/vol) normal donkey serum (Jackson ImmunoResearch Laboratories, West Grove, PA) in PBST for 1 h at room temperature. Different primary antibody solutions were added for incubations at 4 °C overnight, followed by quick PBST washing and reactions (1 h) with the corresponding secondary antibodies (Table 1) at room temperature. After washing, the slides were cover-slipped with Vectorshield^®^ Anti-Fade Mounting Medium with DAPI (Vector Laboratories, Burlingame, CA) for evaluations (see Additional file 2: Table S2 for IHC protocol specifics). In addition, tissue sections of both experimental groups for staining of molecular markers CGRP, p75NTR, serotonin (5HT), and Homer-1a were first processed with antigen retrieval treatment (Additional file 3: Table S3). Briefly, the slides were processed according to Steps 1–5 as described in Additional file 2: Table S2 before incubation at 85 °C for 15 min with tissue sections immersed in the antigen retrieval buffer (i.e., 1× citrate-based antigen unmasking solution; Vector Laboratories, Burlingame, CA). After PBS washing, the slides were subjected to procedures detailed in Steps 6–16 in Additional file 2: Table S2 (see Additional file 3: Table S3 for more details).

**Table 1:** Antibodies for immunohistochemical (IHC) reactions

Antibody	Provider	Host	Address	Dilution
Primary antibodies				
Tumor necrosis factor alpha (TNFα)	ABCam	Mouse	Cambridge, UK	1:200
Inducible nitric oxide synthase (iNOS)	ABCam	Rabbit	Cambridge, UK	1:200
Glial fibrillary acidic protein (GFAP)	ABCam	Goat	Cambridge, UK	1:400
Ionized calcium binding adaptor molecule 1 (Iba1)	Novus Biologicals	Goat	Littleton, CO	1:200
Calcitonin gene-related peptide (CGRP)	ABCam	Mouse	Cambridge, UK	1:400
p75 neurotrophin receptor (p75NTR)	ABCam	Rabbit	Cambridge, UK	1:200
5-Hydroxytryptamine (5HT; Serotonin)	Immunostar	Rabbit	Hudson, WI	1:500
Homer protein homolog 1a (Homer-1a)	Santa Cruz Bio	Goat	Dallas, TX	1:100
Neuronal nuclear protein (NeuN)	EMD Millipore	Mouse	Burlington, MA	1:400
c-Fos: (cellular Fos proto-oncogene or AP-1 transcription factor)	Cell signaling	Rabbit	Danvers, MA	1:300
Brain-derived neurotrophic factor (BDNF)	ABCam	Rabbit	Cambridge, UK	1:200
Glutamic acid decarboxylase 67 (GAD67)	MilliporeSigma	Mouse	St. Louis, MO	1:500
Nestin	Santa Cruz Bio	Mouse	Dallas, TX	1:200
SRY (sex determining region Y)-box 2 (SOX2)	ABCam	Rabbit	Cambridge, UK	1:200
Doublecortin (DCX)	Santa Cruz Bio	Mouse	Dallas, TX	1:200
Secondary antibodies				
Alexa Flour® 488 Donkey Anti-Goat IgG (H+L)	Jackson ImmunoResearch	Donkey	West Grove, PA	1:400
Alexa Fluor® 488 Donkey Anti-Rabbit IgG (H+L)	Jackson ImmunoResearch	Donkey	West Grove, PA	1:400
Alexa Flour® 488 Donkey Anti-Mouse IgG (H+L)	Jackson ImmunoResearch	Donkey	West Grove, PA	1:400
Cy3® Donkey Anti-Rabbit IgG (H+L)	Jackson ImmunoResearch	Donkey	West Grove, PA	1:400
Alexa Fluor® 647 Donkey Anti-Goat lgG (H+L)	Jackson ImmunoResearch	Donkey	West Grove, PA	1:400
Alexa Fluor® 647 Fab Fragment Donkey Anti-Mouse IgG (H+L)	Jackson ImmunoResearch	Donkey	West Grove, PA	1:400

*5-HT* 5-hydroxytryptamine (serotonin), *BDNF* brain-derived neurotrophic factor, *CGRP* calcitonin gene-related peptide, *DCX* doublecortin, *Fos* the FBJ (Finkel–Biskis–Jenkins) murine osteosarcoma virus (v-Fos), *GAD67* glutamic acid decarboxylase 67, *GFAP* glial fibrillary acidic protein, *Iba1* ionized calcium binding adaptor molecule 1, *iNOS* inducible nitric oxide synthase, *NeuN* neuronal nuclear protein, *p75NTR* p75 neurotrophin receptor, *SOX2* SRY (sex determining region Y)-box 2, *TNFα* tumor necrosis factor alpha

For qualitative assessment and semi-quantification of specific IHC signals, confocal imaging was conducted through a Nikon C2 Laser Scanning Confocal Microscope equipped with NIS-Elements Software 4.30.1 (Nikon, Melville, NY). To determine IHC reaction specificity, the orthogonal slices were collected to reconstruct z-stack 3D images of 10–15 μm thickness consisting of 1-μm steps by using NIS-Elements (Nikon). The positive signal threshold range of each marker was attained by averaging the pixel brightness of the weakest positive labeling signals and that of strongest signals against the average background luminance level [14, 26]. Counting of positive IHC pixels for each marker was performed with ImageJ^®^ (NIH) and Photoshop^®^ (Adobe) to compute the percentage of the area that contained qualified signals of each antigen [i.e., immunoreactivity level (IRL)], relative to the entire visual field comparably selected for all samples. Finally, IRLs of the same IHC signals in the immediately adjacent confocal images were also computed to ensure the specificity and consistency of the IHC outcomes.

### Statistical analysis

Statistical computations were done using GraphPad Prism (version 7.0, GraphPad Software, San Diego, CA). Unless otherwise specified, the data of locomotion, incline plane, mVFT, hot plate tests, and histopathology were evaluated by two-way repeated measures ANOVA with Sidak’s post hoc test suggested by the software based on data features. To compare signal area scales (i.e., IRL) of IHC markers between the SCI and laminectomy groups, Student’s *t* test or Mann–Whitney *U* test was used [26]. Statistical linear regression was performed to determine possible correlation relationships between the IRL of CGRP, p75NTR, Homer-1a, and 5-HT and the hypersensitivity threshold determined by the bilateral hot plate test. In this study, values were expressed as mean ± S.E.M. (the standard error of the mean), and statistical significance was set at *p* < 0.05.

## Results

### General physical condition and signs suggestive of spontaneous pain after SCI

All animals lived through the experiment period except for one rat in the SCI group that was euthanatized due to a lower body skin lesion resulting from excessive licking possibly triggered by allodynia (note: another rat was removed from the study due to inadequate injury); light hematuria in several SCI rats resolved within 3–5 days. The group mean time to restore a reflex bladder in SCI rats was 4.3 ± 1.8 days (*n* = 12), which was typical for this model of injury [26] and much shorter than in rats with contusion SCI [27]. Control animals had neither postsurgical complication nor functional deficit. Thus, the group size of SCI and control animals was *n* = 12 and 10, respectively (Fig. 1A, B).

Changes in body weight were recorded to monitor wellbeing of the rats. There was an anticipated degree of body weight loss within the first week following T10 compression. Specifically, relative to the pre-SCI level, the mean percentage of body weight reduction was 0% for the laminectomy group, and 4.2% for the SCI group (range: 0–8.1%; *n* = 12), which was far below 15% set as a criterion to remove animals from the study. All SCI rats recovered their body weight to levels before surgery after the first week p.i., and afterward, continuously gained weight at a rate normally observed in rats following similar surgical procedures (data not shown) [14, 26].

To detect possible presence of spontaneous pain, a central component of clinical NP [5, 7], signs suggestive of stress and/or pain, which included porphyrin staining, reduced grooming, licking or scratching of an intact body area, vocalization, etc., were scored based on housing checking chart records using a formula we developed (see Additional file 1: Table S1 for specifics including references). In the range of 0–38, a score ≥ 5 attained from a non-invoked animal suggested likely presence of pain. Whereas all control rats (*n* = 10) had 0 points during weeks 2–8 p.i., the SCI rats (*n* = 12) showed scores in a range of 5–10 and 2–5 in weeks 2–5 and 6–8, respectively, with the group median score consistently being 5, indicating that some mild degree of pain might exist since the average body weight gains were not disturbed.

### Post-SCI reduction of hindlimb coordinated motor function

#### Locomotion

Evaluation based on the BBB scale revealed marked deficits in hindlimb locomotion in rats after T10 SCI; in contrast, all rats after laminectomy showed no locomotor dysfunction (i.e., BBB score = 21; Fig. 1C). SCI rats demonstrated distinct reduction of hindlimb locomotor ability that was most profound at day 1 p.i. (Fig. 1C). However, compared to the typical sign of spinal shock (i.e., complete flaccid paralysis: BBB score = 0) that occurs during the first 24 h after lower thoracic contusion and T10 severe compression (50 g × 5 min) [26, 27], the SCI rats did not manifest spinal shock syndrome (i.e., group mean BBB score/day-1 p.i.: 3.1 ± 1.4; *n* = 12; Fig. 1C). Thereafter, hindlimbs showed a gradual recovery of locomotion that plateaued 3–4 weeks later. At the end of the study, the SCI group's mean BBB score was 16.1 ± 0.9 points (versus 21 ± 0 of the control group), showing a chronic deficit level that was less severe than that observed in the previous study using the same compression regimen (i.e., BBB scores of ~ 12) [14].

#### Incline plane test

Compared to the control group, the mean maximum degree where SCI rats could stabilize their body postures while facing-downward, mainly by the hindlimbs was significantly lower in 1 day p.i. (37.6 ± 2.0°/SCI versus a normal range of 55.7 ± 0.8°/control, *p* < 0.05; two-way repeated measures ANOVA with Sidak’s post hoc test). As seen in the locomotor changes, incline plane performance of the SCI rats improved more over the first 3 weeks before plateauing around 4–8 weeks p.i. (Fig. 1D). At 8 weeks p.i., the SCI group’s mean maximum incline plane degree was 49.3 ± 1.3°, which remained significantly lower than the 57.2 ± 0.9° of the control group (*n* = 12/SCI or 10/control; *p* < 0.05 to < 0.001; two-way repeated measures ANOVA with Sidak’s post hoc test).

In this study, the moderate T10 compression formula resulted in group average BBB scores and incline plane angles that were much closer to that of mild compression (20 g × 5 min) reported earlier [14, 26]. Thus, the term of “moderate compression” was used to specify the physical regimen of the quasi-static compression per se (35 g × 5 min). This discrepancy was probably caused by subtle differences in surgical details such as laminectomy size produced by different operation performers.

### Post-SCI abnormalities of sensory functions

#### Neurological reflexes

The contact righting reflex was evaluated to assess the ability of the spinal cord to coordinate with the brainstem, peripheral afferent and efferent system, and neuromuscular junctions to correct abnormal body postures [14, 26, 33–35]. Furthermore, postural (placing) and spinal reflexes were tested [14, 26]. These data were presented as the percentage of rats in either group that had normal reflexes [14].

All SCI rats lost the ability to perform normal righting reflex (Fig. 2A), proprioceptive positioning reflex of paw placing (Fig. 2B), and withdrawal response to hindlimb extension (Fig. 2C), at 1 day p.i. There were noticeable recoveries of the three categories of reflexes in the following weeks with 80% SCI rats showing normal righting and paw placing reflexes (Fig. 2A, B), and extension reflex (Fig. 2C) by 3 and 4 weeks p.i., respectively. Conversely, withdrawal reflexes induced by brief pressuring (i.e., pressure withdrawal reflex; Fig. 2D) and pinching (i.e., nociception withdrawal reflex; Fig. 2E) of the hindpaw were detectable (despite severe reductions) 1 day p.i. (i.e., no spinal shock). Their recovery rates, however, were much slower and incomplete in comparison to those of the righting (Fig. 2A), placing (Fig. 2B), or extension reflex (Fig. 2C). For example, by 8 weeks p.i., only ~ 33% rats with SCI showed a normal nociception withdrawal reflex (Fig. 2E), which was much lower than that of righting, placing, or extension reflex recovery (i.e., 100%; Fig. 2A–C), revealing a protracted abnormality of nociceptive signal-induced neurological reflexes in rats with T10 SCI.

#### Mechanical hypersensitivity

Mechanical allodynia, a representative symptom of NP, is a hypersensitivity triggered by innocuous stimuli similar to light touch. Clinically, the standardized VFT is used to detect a mechanical sensory threshold in the quantitative sensory testing (QST) for pain in patients [36]. In this study, mVFT, which emulated light touch, was performed to detect evoked mechanical hypersensitivity (Fig. 2F) [14, 37]. Before SCI, the group withdrawal sensitivity threshold averaged 8.2 ± 0.6 g/control and 7.5 ± 0.4 g/SCI in the forepaws (*p* > 0.05; Fig. 2G), and 8.6 ± 0.4 g/control and 9.0 ± 0.8 g/SCI in the hindpaws (*p* > 0.05; Student’s *t* test; Fig. 2H). In contrast to a stable array of sensitivity thresholds attained from the control group, at day 1 p.i., the mean withdrawal sensitivity threshold drastically increased to 11.1 ± 1.5 g and 45.8 ± 5.1 g in the forepaws and hindpaws of SCI rats, respectively (Fig. 2G, H), suggesting that there was severe loss of neurological functions close to that of spinal shock. Thereafter, the group mean withdrawal threshold, compared to the pre-SCI level and control values, significantly decreased in the forepaws (i.e., above-injury level allodynia), starting 2 weeks after SCI (4.7 ± 0.7 g/week 2 p.i., *p* < 0.05; 3.9 ± 0.5 g/week 5 p.i., *p* < 0.01; and 3.8 ± 0.9 g/week 8 p.i., *p* < 0.01; *n* = 12; Fig. 2G); the hindpaws of SCI animals also had significantly decreased mean withdrawal sensitivity threshold (i.e., below-injury level allodynia; 4.1 ± 0.6 g/week 2 p.i., *p* < 0.01; 3.6 ± 0.4 g/week 5 p.i., *p* < 0.01; and 4.0 ± 0.3 g/week 8 p.i., *p* < 0.01; *n* = 12; Fig. 2H). Lastly, at-injury level mechanical allodynia measured in the dorsal dermatomes adjacent to the T10 injury epicenter (Fig. 2I) exhibited a comparable pattern of significant augmentation of hypersensitivity, compared to the control group (0.8 ± 0.3 g/week 2 p.i., *p* < 0.01; 0.5 ± 0.2 g week 5 p.i., *p* < 0.01; and 0.4 ± 0.1 g week 8 p.i., *p* < 0.01; *n* = 12/SCI or 10/control; two-way repeated measures ANOVA with Sidak’s post hoc test; Fig. 2I). For all three levels tested, there were no significant changes in withdrawal sensitivity thresholds in the laminectomy control group. These data demonstrated that SCI rats displayed subacute and chronic hypersensitivities to mechanical stimuli above-, below-, and at-injury level.

#### Thermal hypersensitivity

Thermal hypersensitivity threshold is an important parameter in the QST for diagnosing thermal allodynia, another important symptom of clinical SCI NP [38–41]. SCI and control rats first underwent the bilateral hot plate test for which the pre-SCI and pre-laminectomy mean hindlimb latency was 8.8 ± 0.8 s (*n* = 12) and 8.0 ± 0.9 s (*n* = 10; *p* > 0.5, Student’s *t* test), respectively (i.e., baseline values; Fig. 2J). While the control group showed a steady mean response latency, the group mean latency increased dramatically to 19.1 ± 3.1 s in the injured rats at day 1 p.i., relative to the baseline value (i.e., 8.8 ± 0.8 s), indicating a transient severe reduction of neurological function during the first 24 h p.i. Subsequently, the group mean latency of SCI animals gradually decreased, with the average threshold becoming significantly lower (i.e., ↑thermal hypersensitivity) than the baseline values and those of the control group, starting in week 3 p.i. (Fig. 2J; *n* = 12/SCI or 10/laminectomy; *p* < 0.05; two-way repeated measures ANOVA with Sidak’s post hoc test).

In the more sensitive unilateral hindlimb hot plate test [39], although an overall resemblance to the bilateral hot plate test data was observed, this test detected significantly reduced group mean latencies 1 week earlier, starting day 14 p.i., which lasted till the end of the study, compared to the control group (Fig. 2K; *n* = 12/SCI or 10/control; *p* < 0.05; two-way repeated measures ANOVA with Sidak’s post hoc test). Furthermore, the SCI group mean threshold of this test trended even lower than the bilateral hot plate test results (Fig. 2K), confirming the test was more sensitive.

### Histopathological impacts of moderate T10 compression

**Fig. 3:**
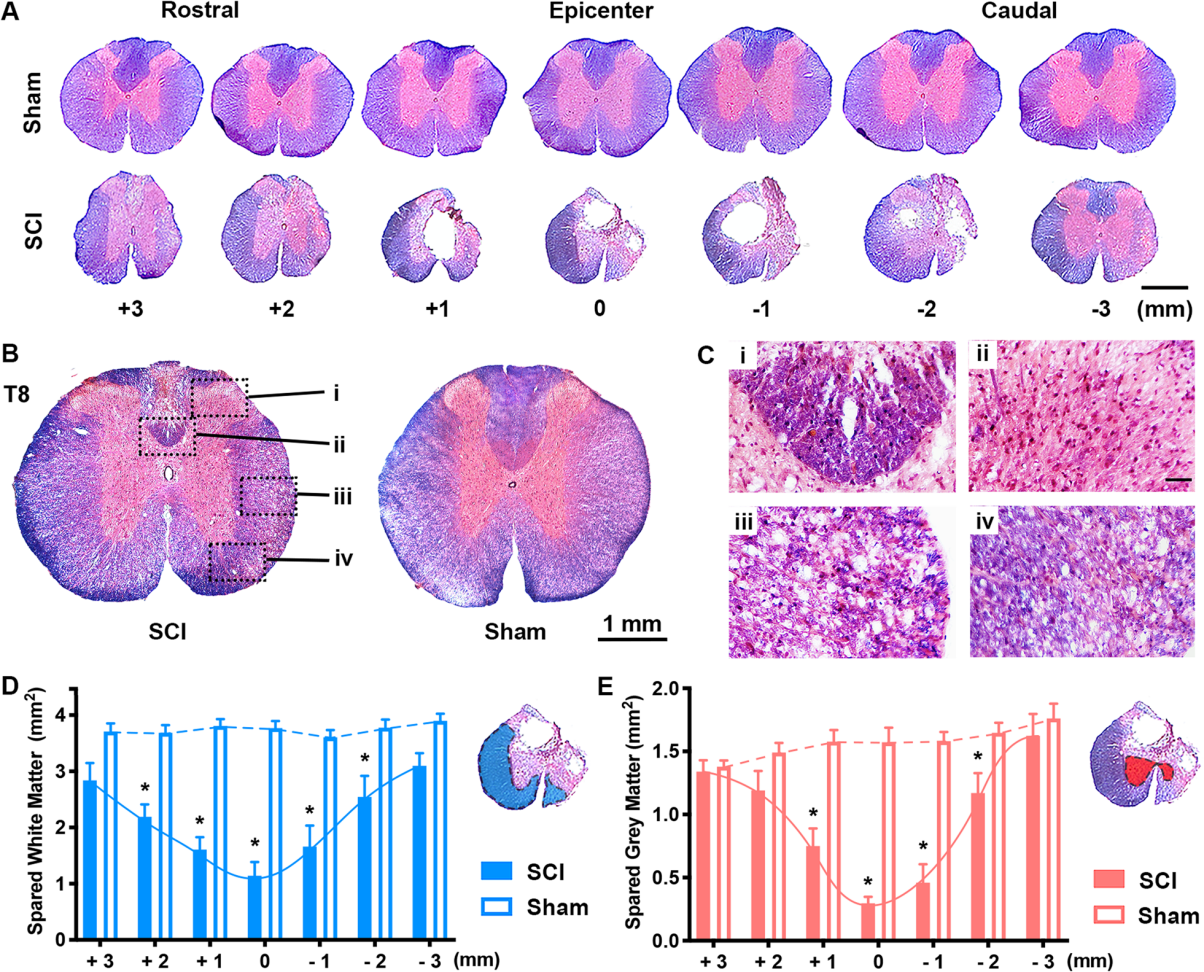
Histopathological outcomes. **A** Coronal spinal cord sections in 20 µm thickness post solvent blue/H&E stain showed histopathological defects in the SCI group only (scale bar: 1 mm). Injured spinal cord sections at and around the epicenter exhibited damages in the white matter and gray matter (e.g., cavities and lesion areas). The injured tissues also contained a large number of basophilic inflammatory cells (stained by hematoxylin) and intra-cavity loose webs of non-neural tissues. **B**, **C** T8 spinal cord sections that were ~ 5 mm rostral to T10 injury site (**B**; framed areas were magnified in **C**), had apparent demyelination (i.e., hypostained by solvent blue) and infiltration of inflammatory cells in both white matter and gray matter (**C**). **D**, **E** Area measurement of representative coronal sections at the injury site (i.e., 0 mm) and 1–3 mm rostral and caudal to it, revealed that compared to the control group, T10 compression significantly reduced the mean residual white matter (**D**) and gray matter areas (**E**) at the epicenter, and in spinal cord loci 1 and 2 mm adjacent to it (*p* < 0.05; two-way repeated measures ANOVA with Sidak’s post hoc test; *n* = 7/group)

Whereas no discernible lesions were found in the laminectomy control tissues, all injured spinal cords exhibited apparent damages in the white matter (dorsal, lateral, and ventral funiculi) and gray matter (dorsal and ventral horns, and intermediate and central regions) at the injury epicenter, forming cavities and lesion areas that further extended rostrally and caudally to demarcate the lesion volume (Fig. 3A). Epicenter coronal sections also contained a high number of basophilic inflammatory cells (i.e., subcellular structures containing nucleic acids stained dark blue/violet by hematoxylin) and intra-cavity loose webs of non-neural tissues (Fig. 3A). Farther away from the epicenter, sections sampled from T8–9 spinal cord that was ~ 5 mm rostral to the compression site (Fig. 3B), exhibited discernible demyelination (i.e., hypostained by solvent blue) and infiltration of inflammatory cells in both the white matter and gray matter (Fig. 3C).

Measurements of digital images of representative coronal sections at the injury site and 1–3 mm rostral and caudal to it, demonstrated that relative to the control group, T10 compression significantly reduced the mean residual white matter (Fig. 3D) and gray matter areas (Fig. 3E) at the epicenter, and in loci 1 and 2 mm adjacent to it (*p* < 0.05; two-way repeated measures ANOVA with Sidak’s post hoc test; *n* = 7/group). Taken together, the morphological evidence showed chronic neuroparenchyma loss and lesions, and presence of inflammatory cells in the epicenter and regions bidirectionally adjacent to it after T10 compression [14, 26].

### Chronic NIF, NTM, NML, and NPL changes at multiple nuclei/regions of the CNS in rats with SCI NP

Our previous work demonstrated that lumbar and cervical NIF co-existed with NP-like behaviors in the early chronic phase of SCI [14, 26]. However, whether NIF after a T10 injury may extend into other thoracic cord levels such as T8 where more sympathetic preganglionic neurons reside [27], and different brain centers involved in pain signaling relay and regulation remained to be systematically investigated [14, 17, 42, 43].

#### Changes of NIF, NTM, NML, and NPL markers in injured spinal cords

**Fig. 4:**
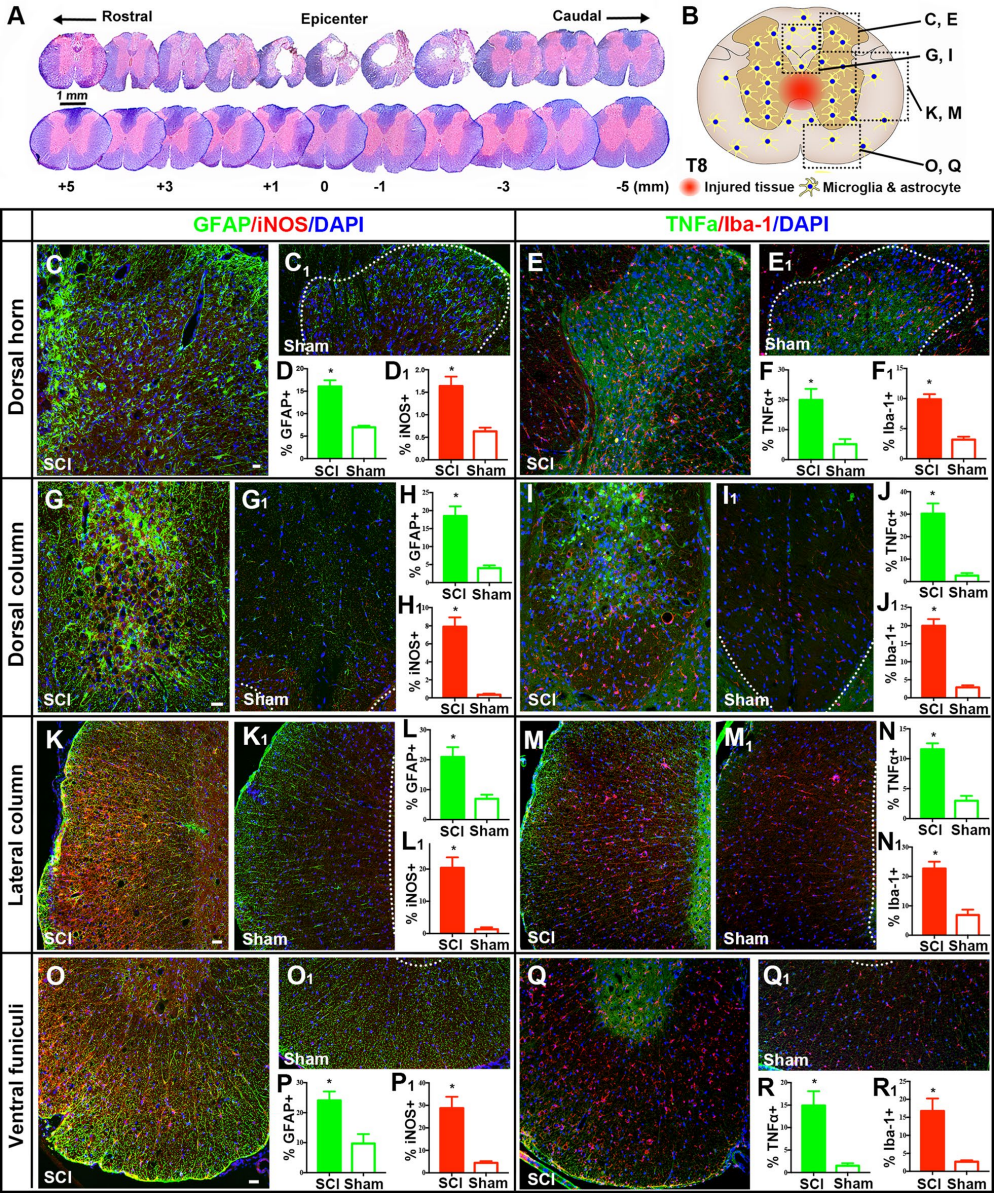
Neuroinflammation in spinal cord tissue rostral to the epicenter. **A** Immunoreactivity level (IRL) of GFAP (reactive astrogliosis), Iba-1/(microglia/macrophage activation), TNF-α (proinflammatory cytokine), and/or iNOS (marker of proinflammatory microglia/macrophage; inflammation mediator) expressions were evaluated in coronal sections sampled from T8 spinal cord (SCI group) or T10 level (control group). **B** Specific areas in each section that were examined by IHC assay (for specific data point, please see individual images accordingly labeled in **C**–**Q**). In the dorsal horn (DH; **C**–**F**_**1**_) and dorsal column (DC; **G**–**J**_**1**_), there were significantly increased expressions of GFAP (DH: **C**, **D**; DC: **G**, **H**) and iNOS (DH: **C**, **D**_**1**_; DC: **G**, **H**_**1**_) in injured spinal cords, compared to the control tissue (DH: **C**_**1**_; DC: **G**_**1**_). Also significantly heightened in the dorsal spinal cord were expressions of TNFα (DH: **E**, **F**; DC: **I**, **J**) and Iba-1 (DH: **E**, **F**_**1**_; DC: **I**, **J**_**1**_; controls: **E**_**1**_/DH and **I**_**1**_/DC; *n* = 4/group; *p* < 0.01; Student’s *t* test). In addition, compared to control sections, the lateral column (LC) and ventral funiculi (VF) of SCI tissues showed significant IRL augmentations of GFAP (LC: **K**, **L**; VF: **O**, **P**), iNOS (LC: **K**, **L**_**1**_; VF: **O**, **P**_**1**_), TNFα (LC: **M**, **N**; VF: **Q**, **R**), and Iba-1 (LC: **M**, **N**_**1**_; VF: **Q**, **R**_**1**_; controls: **K**_**1**_, **M**_**1**_ for LC and **O**_**1**_, **Q**_**1**_ for VF; *n* = 4/group; *p* < 0.01; Student’s *t* test; scale bars: 40 µm/**C**, **G**; 60 µm/**K**, **O**)

To assess NIF responses in T8 neuroparenchyma, IRL of GFAP (astrocyte activation and reactive astrogliosis), Iba-1 (microglia/macrophage activation), TNFα (proinflammatory cytokine), and/or iNOS (proinflammatory polarization marker of microglia/macrophages and an inflammation mediator) expressions were evaluated in spinal cord sections sampled from 5 mm rostral to the injury epicenter (SCI group) or T10 level (i.e., laminectomy site; control group) (Fig. 4A; see Fig. 4B for the labeling of the framed areas that were examined: corresponding higher magnification images were in Fig. 4C–Q). Both the dorsal horn (DH) and dorsal column (DC) had significantly increased expression of GFAP (DH: Fig. 4C, D; DC: Fig. 4G, H) and iNOS (DH: Fig. 4C, D_1_; DC: Fig. 4G, H_1_) in injured spinal cords, compared to the control tissue (DH: Fig. 4C_1_; DC: Fig. 4G_1_). Also significantly elevated in injured spinal cords were expressions of TNFα (DH: Fig. 4E, F; DC: Fig. 4I, J) and Iba-1 (DH: Fig. 4E, F_1_; DC: Fig. 4I, J_1_; controls: Fig. 4E_1_/DH and Fig. 4I_1_/DC; *n* = 4/group; *p* < 0.01; Student’s *t* test). In addition, compared to the control group, the lateral column (LC) and ventral funiculi (VF) of SCI tissues showed significant IRL augmentations of GFAP (LC: Fig. 4K, L; VF: Fig. 4O, P), iNOS (LC: Fig. 4K, L_1_; VF: Fig. 4O, P_1_), TNFα (LC: Fig. 4M, N; VF: Fig. 4Q, R), and Iba-1 (LC: Fig. 4M, N_1_; VF: Fig. 4Q, R_1_; controls: Fig. 4K1, M_1_ for LC/Fig. 4O_1_, Q_1_ for VF; *n* = 4/group; *p* < 0.01; Student’s *t* test).

**Fig. 5:**
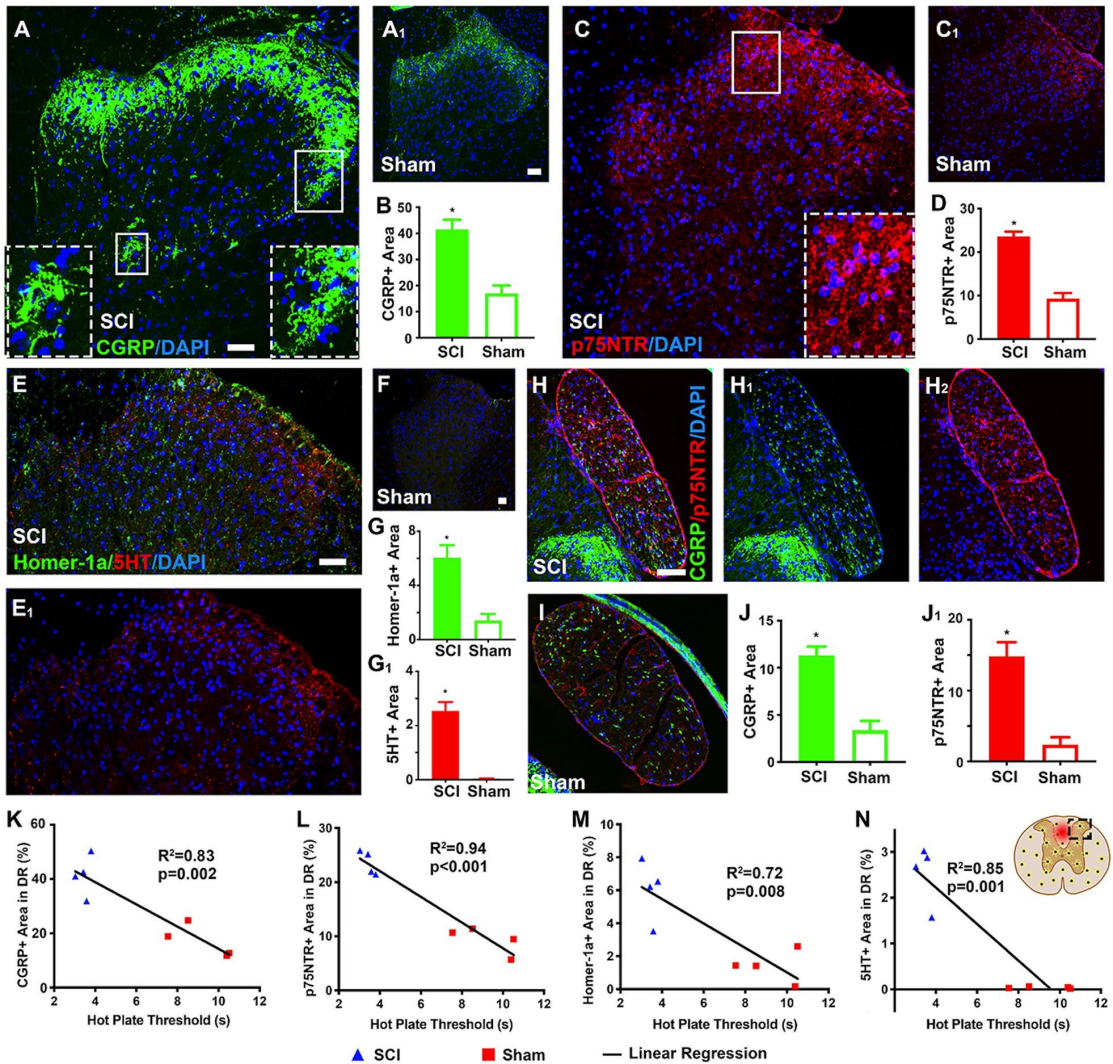
Changes of neurotransmission, neuroplastic, and neuromodulatory molecules in injured spinal cords. IRL of calcitonin gene-related peptide (CGRP, a pain-related neurotransmitter) and p75 neurotrophin receptor (p75NTR, a regulator of nociceptive neurons) were significantly higher in spinal cord sections 5 mm rostral to the epicenter (i.e., ~ T8 spinal cord; SCI group; **A** and **B**/CGRP; **C** and **D**/p75NTR), compared to T10 level of the control (**A**_**1**_/CGRP; **C**_**1**_/p75NTR); the IHC signals were primarily located in Rexed Laminae (RL) I and II (CGRP: right inset in **A**; p75NTR: inset in **C**) and deeper zones of DH (CGRP: left inset in **A**) and in the dorsal roots (CGRP: **H**_**1**_, **J**; p75NTR: **H**_**2**_, **J**_**1**_; **I**/control; *p* < 0.01, *n* = 4/group, Student’s *t* test). Also disclosed by IHC analysis were significantly increased mean IRL of Homer-1a, a marker of DH neuronal plasticity (**E**, **G**) and serotonin (5HT), a neuromodulator (**E**_**1**_, **G**_**1**_) in RL-I & II of the SCI group, relative to the control group (**F**; *p* < 0.05, *n* = 4/group, Student’s *t* test). Furthermore, statistical linear regressions uncovered that IRL of CGRP and p75NTR were negatively correlated with the sensitivity threshold (unit: second) of the bilateral hot plate test (CGRP: *p* = 0.02, *R*^2^ = 0.83, **K**; p75NTR: *p* < 0.01, *R*^2^ = 0.94, **L**) in the SCI group; changes in IRL of Homer-1α and 5HT also correlated negatively with the sensitivity threshold of the bilateral hot plate test in SCI animals (Homer-1α: *p* < 0.01, *R*^2^ = 0.72, **M**; 5HT: *p* < 0.01, *R*^2^ = 0.85, **N**; scale bars: 100 µm)

Next, expression levels of calcitonin gene-related peptide (CGRP), a main neurotransmitter released from the C and Aδ sensory fiber terminals onto the substantia gelatinosa neurons, which facilitates NP/pain development [44, 45], and p75 neurotrophin receptor (p75NTR), a key receptor to regulate peripheral and central nociceptive neurons [46], were evaluated with IHC assays of T8 spinal cord sections (SCI group) or at T10 level (control). Relative to control tissues (Fig. 5A_1_/CGRP; Fig. 5C_1_/p75NTR), significantly heightened group mean IRL of CGRP (Fig. 5A, B) and p75NTR (Fig. 5C, D) were detected primarily in Rexed Laminae (RL) I and II (CGRP: *right* inset in Fig. 5A; p75NTR: inset in Fig. 5C), deeper zones of DH (CGRP: *left* inset in Fig. 5A), and the dorsal roots of SCI tissues (CGRP: Fig. 5H_1_, J; p75NTR: Fig. 5H_2_, J_1_; Fig. 5I/control; *p* < 0.01, *n* = 4/group, Student’s *t* test). For SCI NP-triggered NPL and NML alterations [14], IHC analysis uncovered significantly increased group mean IRL of Homer-1a, a molecular marker for DH neuronal plasticity (Fig. 5E, G) [14] and serotonin (5HT, a neuromodulator; Fig. 5E_1_, G_1_) in RL-I and II, relative to the control group (Fig. 5F; *p* < 0.05, *n* = 4/group, Student’s *t* test).

Lastly, to determine possible functional impacts associated with expression changes of the afore-described molecules p.i., statistical linear regressions were conducted. The results showed that IRL of CGRP and p75NTR were negatively correlated with the thermosensitivity threshold (unit: second) determined by the bilateral hot plate test (CGRP: *p* = 0.02, *R*^2^ = 0.83, Fig. 5K; p75NTR: *p* < 0.01, *R*^2^ = 0.94, Fig. 5L) for the SCI group. Moreover, changes in IRL of Homer-1α and 5HT also correlated negatively with the thermosensitivity threshold derived from the bilateral hot plate test in animals with SCI (Homer-1α: *p* < 0.01, *R*^2^ = 0.72, Fig. 5M; 5HT: *p* < 0.01, *R*^2^ = 0.85, Fig. 5N). These data suggested that increased DH expressions of CGRP and p75NTR might have worsened SCI NP, which could have triggered stronger NPL and NML responses (e.g., higher expressions of Homer-1α and 5HT, respectively, in RL-I and II of DH).

#### Chronic NIF, neuronal hyperactivity, and serotonin reduction in the brainstem

**Fig. 6:**
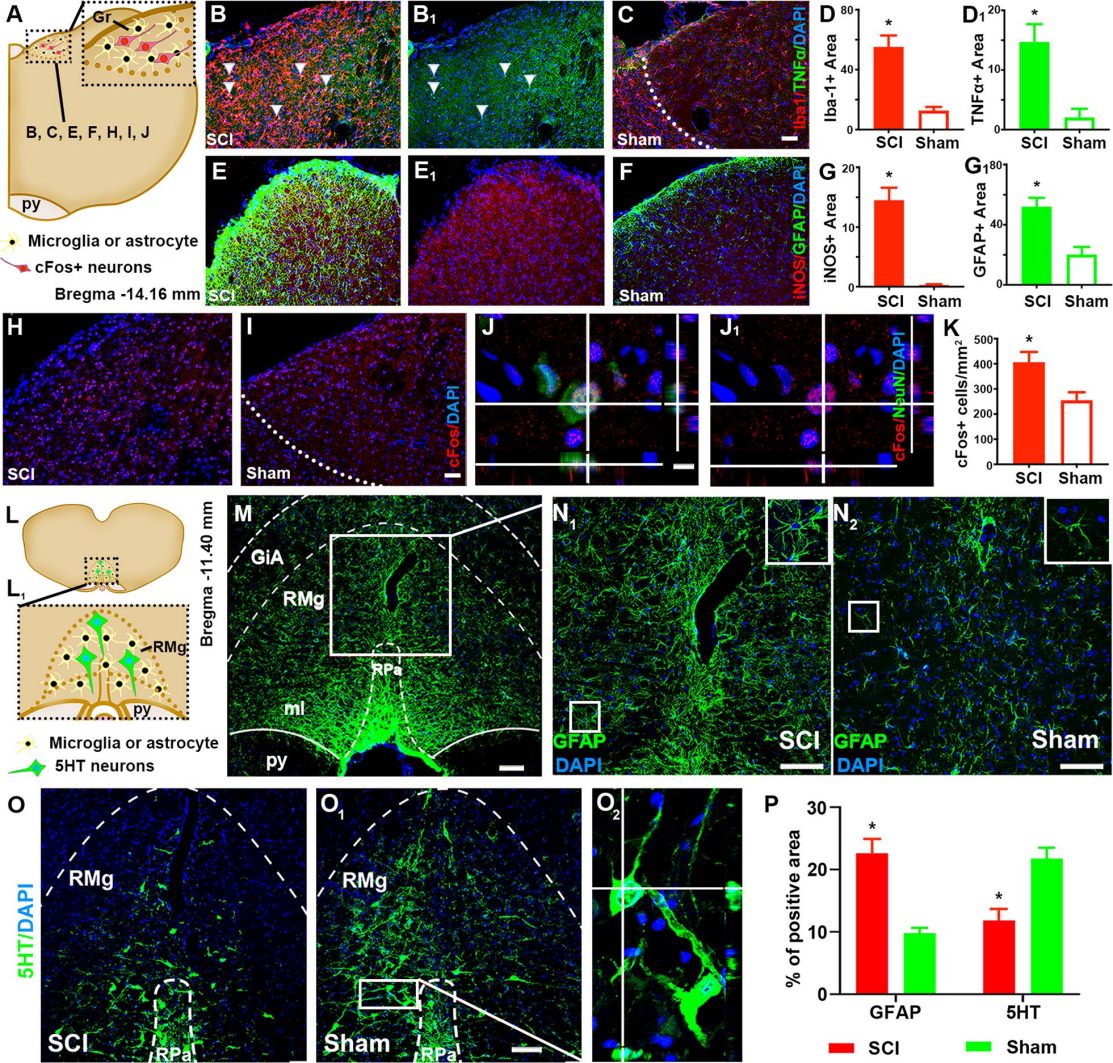
Chronic neuroinflammation, neuronal hyperactivity, and serotonin reduction in the brainstem. Neurons in the gracile nucleus (GrN; **A**) of SCI rats had significantly higher group mean IRL of Iba-1 (**B**, **D**) and TNFα (**B**_**1**_, **D**_**1**_), compared to those of control animals (**C**). IHC double stains revealed significantly elevated mean IRL of GFAP (**E**, **G**_**1**_) and iNOS (**E**_**1**_, **G**) in the SCI group relative to the control group (**F**; *n* = 4/group; *p* < 0.05, Student’s *t* test). The number of neurons expressing c-Fos, a neuronal activity marker in the GrN of SCI animals was significantly higher (**H** versus **I**) than that of controls (**K**, *n* = 4/group; *p* < 0.05, Student’s *t* test). Under the orthogonal slicing, co-localization of cFos and NeuN, a highly specific marker of neuronal nuclei (**J**) and DAPI, a standard nuclear counterstain (**J**_**1**_; scale bars: 100 µm/**C** and **I**; 40 µm/**J**) was confirmed. In the nucleus raphe magnus (RMg or NRM; **L**), a serotonergic neuronal center (**L**_**1**_, **M**), significantly increased group average IRL of GFAP was observed in the SCI group, compared to the control group (**N**_**1**_ versus **N**_**2**_). GFAP was mainly produced by hypertrophic astrocytes in injured spinal cords (inset image in **N**_**1**_ versus that of **N**_**2**_). In contrast, SCI NRM had discernibly lower group mean IRL of 5-HT expression (**O**) than that of the control NRM (**O**_**1**_; statistics in **P**; *p* < 0.001/GFAP and 0.01/5HT; Student’s *t* test; *n* = 5/group). Confocal z-stack imaging with orthogonal optical slicing captured typical morphology of serotonergic neurons (i.e., 5-HT presence in beaded axons and cell soma: **O**_**2**_; scale bars: 100 µm/**M**–**O**_**1**_)

Situated in the medulla oblongata at the junction of the cervical spinal cord and brainstem, neurons in the GrN (Fig. 6A) relay somatosensory information from the lower half of the body and the legs to the thalamus and participate in maintaining NP [47, 48]. In this study, significantly higher group mean IRL of Iba-1 (Fig. 6B, D) and TNFα (Fig. 6B_1_, D_1_) presented in brainstem coronal sections containing GrN of SCI rats compared to those of the control group (Fig. 6C). IHC double stains of the same level sections showed significantly elevated mean IRL of GFAP (Fig. 6E, G_1_) and iNOS (Fig. 6E_1_, G) in the SCI rats relative to the control group (Fig. 6F; *n* = 4/group; *p* < 0.05, Student’s *t* test). Because c-Fos expression in secondary sensory neurons in the GrN could be augmented by electrical or nerve injury stimulation of c-fibers to emulate nociceptive afferent signals [49], numbers of cFos+ neurons in GrN were quantified via IHC co-localization of cFos, and nuclear markers of NeuN and DAPI. The mean number of neurons producing cFos was significantly higher in SCI animals than that of controls (Fig. 6H/SCI versus Fig. 6I/control; statistics in Fig. 6K, *n* = 4/group; *p* < 0.05, Student’s *t* test). Orthogonal slicing confirmed co-localization of cFos, NeuN, a highly specific marker of neuronal nuclei (Fig. 6J), and DAPI, a standard nuclear counterstain (Fig. 6J_1_).

Positioned in the rostral ventromedial medulla (RVM) of the brainstem is the nucleus raphe magnus (NRM or RMg; Fig. 6L), a serotonergic nucleus (Fig. 6L_1_, M) engaged in nociception/pain regulation. The NRM receives inputs from the PAG (see below) and mainly projects to the spinal cord DH [50]. In the SCI tissue sections, significantly increased group average IRL of GFAP was observed in NRM, compared to the control group (Fig. 6N_1_/SCI versus Fig. 6N_2_/control; note: GFAP was mainly produced by hypertrophic astrocytes in injured spinal cords; comparing inset image in Fig. 6N_1_ to that of Fig. 6N_2_). In contrast, SCI NRM had markedly reduced group average IRL of 5-HT expression (Fig. 6O) relative to that of the control group (Fig. 6O_1_; statistics in Fig. 6P; *p* < 0.001/GFAP or 0.01/5HT; Student’s *t* test; *n* = 5/group). Notably, orthogonal optical slicing of confocal z-stack images captured typical morphology of serotonergic neurons (i.e., 5-HT presence in the soma cytoplasm and axonal varicosities of the cells; Fig. 6O_2_). The decrease of 5HT expression in the serotonergic neurons suggested that T10 compression induced regional/systemic NIF and heightened DH demand of 5HT (Fig. 5E_1_, G_1_), which might conjointly diminish NRM production and storage of 5HT.

**Fig. 7:**
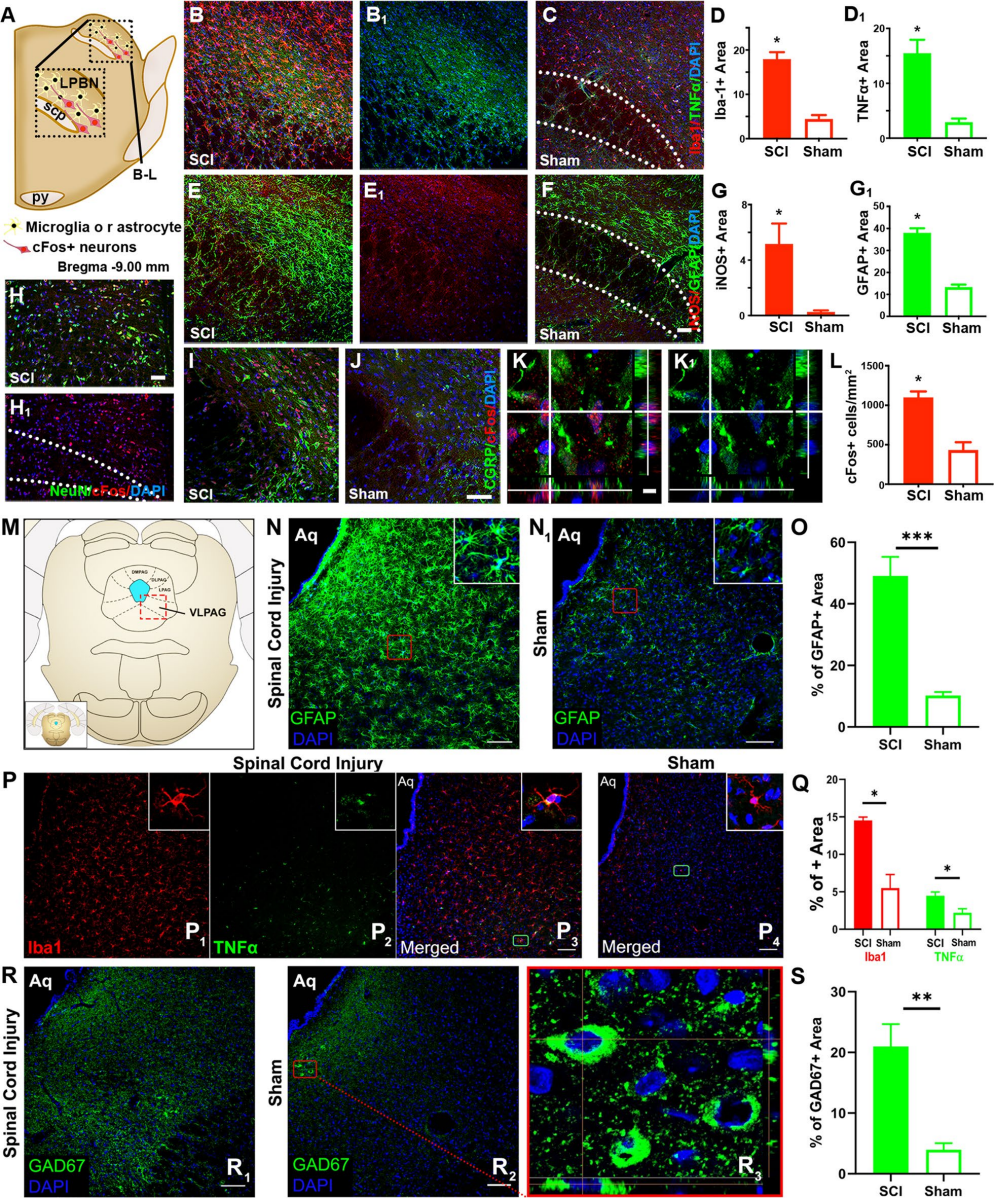
Chronic neuroinflammation and neurotransmitter changes in the pons and midbrain. The lateral parabrachial nuclei (LPBN; **A**; scp: superior cerebellar peduncle) displayed significantly increased mean IRL of co-stained Iba-1 (**B**, **D**)/TNFα (**B**_**1**_, **D**_**1**_) and iNOS (**E**_**1**_, **G**)/GFAP (**E**, **G**) in SCI tissues, compared to control tissues (Iba-1/TNFα: **C**; iNOS/GFAP: **F**; *n* = 4/group, *p < 0.05, Student’s *t* test). IHC images showed morphological features of aggregation of activated microglia and astrocytes in LPBN after SCI, corroborating with increased detection of Iba-1, TNFα and iNOS, and GFAP in these cells (**B**, **B**_**1**_, **E**_**1**_, and **E**, relative to controls in **C** and **F**). T10 compression heightened the activity of NeuN+ (**H**)/CGRP+ (**I**) neurons in the LPBN compared to the control group (**J**; confocal analysis in **K**/SCI and **K**_**1**_/control), which was demonstrated by significantly higher numbers of cFos+ cells co-expressing CGRP in SCI LPBN (statistics in **L**; *n* = 4/group, *p < 0.05, Student’s *t* test). The ventral lateral periaqueductal gray (VLPAG: Bregma −7.80 mm; **M**) in the midbrain of the SCI group showed higher mean IRL of GFAP and Iba1/TNFα co-staining (**N** and **P**_**1**_**/P**_**2**_**/P**_**3**_, respectively) than the control group (**N**_**1**_/GFAP; **P**_**4**_/Iba1 and TNFα); the difference was statistically significant (**O**/GFAP, *n* = 4/group, ***p < 0.001; Student’s *t* test; **Q**/Iba1 and TNFα, *p < 0.05; Mann–Whitney *U* test; *n* = 4/group). Under higher magnification, SCI VLPAG manifested extensive reactive gliosis (e.g., morphology of hypertrophic astrocytes: **N** inset), relative to the control tissue (**N**_**1**_ inset). Activated microglia (i.e., swollen ramified morphology and expressions of Iba1/**P**_**1**_ inset and TNFα/**P**_**2**_ inset (merged images: **P**_**3**_ inset) densely populated VLPAG of SCI animals only (relative to laminectomy controls: **P**_**4**_ and inset). Importantly, discernably elevated GAD67 expression scale existed in the VLPAG of the SCI group (**R**_**1**_) than the control group (**R**_**2**_). Confocal z-stack imaging with orthogonal optical slicing depicted a typical profile of GABAergic neurons with GAD67 immunostaining in the neuronal somas and neurites (**R**_**3**_). The mean IRL of GAD67 in the SCI VLPAG was significantly higher than that of the control group (**S**; *n* = 5/group, **p < 0.01; Student’s *t* test; scale bars: 100 µm/**N**–**R**_**2**_)

Farther rostral to GrN and NRM, located bilaterally in the dorsolateral pons, are the lateral PBN (LPBN: Fig. 7A) that disseminate sensory information (e.g., temperature, pain, etc.) to forebrain structures (e.g., the thalamus, hypothalamus, and extended amygdala) with the involvement of their CGRP+ neurons in the development of NP [50–52]. SCI LPBN displayed significantly elevated group mean IRL of co-stained Iba-1 (Fig. 7B, D)/TNFα (Fig. 7B_1_, D_1_) and iNOS (Fig. 7E_1_, G)/GFAP (Fig. 7E, G), compared to control LPBN (Iba-1/TNFα: Fig. 7C; iNOS/GFAP: Fig. 7F; *n* = 4/group, *p* < 0.05, Student’s *t* test). Furthermore, IHC images showed typical morphological features of activation and aggregation of microglia and astrocytes in the LPBN area after SCI, corroborating with increased IHC detection of Iba-1, TNFα and iNOS, and GFAP in these cells (Fig. 7B, B_1_, E_1_, and E, relative to Fig. 7C, F), respectively. SCI boosted the activity of NeuN+ (Fig. 7H)/CGRP+ neurons in the LPBN (i.e., cFos+/CGRP+/DAPI+ cells in Fig. 7I) compared to the control group (Fig. 7J; confocal analysis in Fig. 7K/SCI and Fig. 7K_1_/control): there were significantly more cFos+ neurons co-expressing CGRP in the SCI LPBN tissue (Fig. 7I versus J; statistics in Fig. 7L; *n* = 4/group, *p* < 0.05, Student’s *t* test). This data suggested that pathophysiological activation of the LPBN CGRP neurons co-existed with NIF and NP in rats after T10 compression.

#### Chronic NIF and higher GAD67 level in GABAergic neurons in the PAG

The PAG (also termed the central gray), especially the ventral lateral PAG (VLPAG: Fig. 7M) in the midbrain, is a major descending modulation center of pain [53]. Here, the VLPAG region of the SCI rats showed higher mean IRL of GFAP and Iba1/TNFα co-staining (Fig. 7N, P_1–3_, respectively) than the control group (Fig. 7N_1_/GFAP; Fig. 7P_4_/Iba1 and TNFα double-staining); the difference was statistically significant (Fig. 7O/GFAP, *n* = 4/group, *p* < 0.001; Student’s *t* test; Fig. 7Q/Iba1 and TNFα, *p* < 0.05; Mann–Whitney *U* test; *n* = 4/group). SCI VLPAG displayed extensive reactive gliosis (e.g., morphologically hypertrophic astrocytes: Fig. 7N inset), relative to the control tissue (Fig. 7N_1_ inset). Additionally, activated microglia that exhibited swollen ramified morphology and expression of Iba1 (Fig. 7P_1_ inset) and TNFα (Fig. 7P_2_ inset; merged images: Fig. 7P_3_ inset), were found to have densely populated VLPAG only in SCI animals (versus laminectomy controls in Fig. 7P_4_ and inset). Finally, there was discernably more GAD67 expression in GABAergic neurons [i.e., neurons that produced gamma-aminobutyric acid (GABA), which is the main inhibitory neurotransmitter in the mammalian CNS] in the VLPAG of the SCI group (Fig. 7R_1_) compared to the control group (Fig. 7R_2_). Orthogonal optical slicing depicted a typical morphological profile of GABAergic neurons with GAD67 immunostaining in the neuronal somas and neurites (Fig. 7R_3_). Indeed, the mean IRL of GAD67 in the SCI VLPAG was significantly higher than that of the control group (Fig. 7S; *n* = 5/group, *p* < 0.01; Student’s *t* test), suggesting that NIF might have induced augmentation of neuronal GAD67 (i.e., ↑GABA) in the PAG to suppress descending pain inhibition mechanisms.

#### Chronic NIF and altered NSC activity in the subcortical structures

**Fig. 8:**
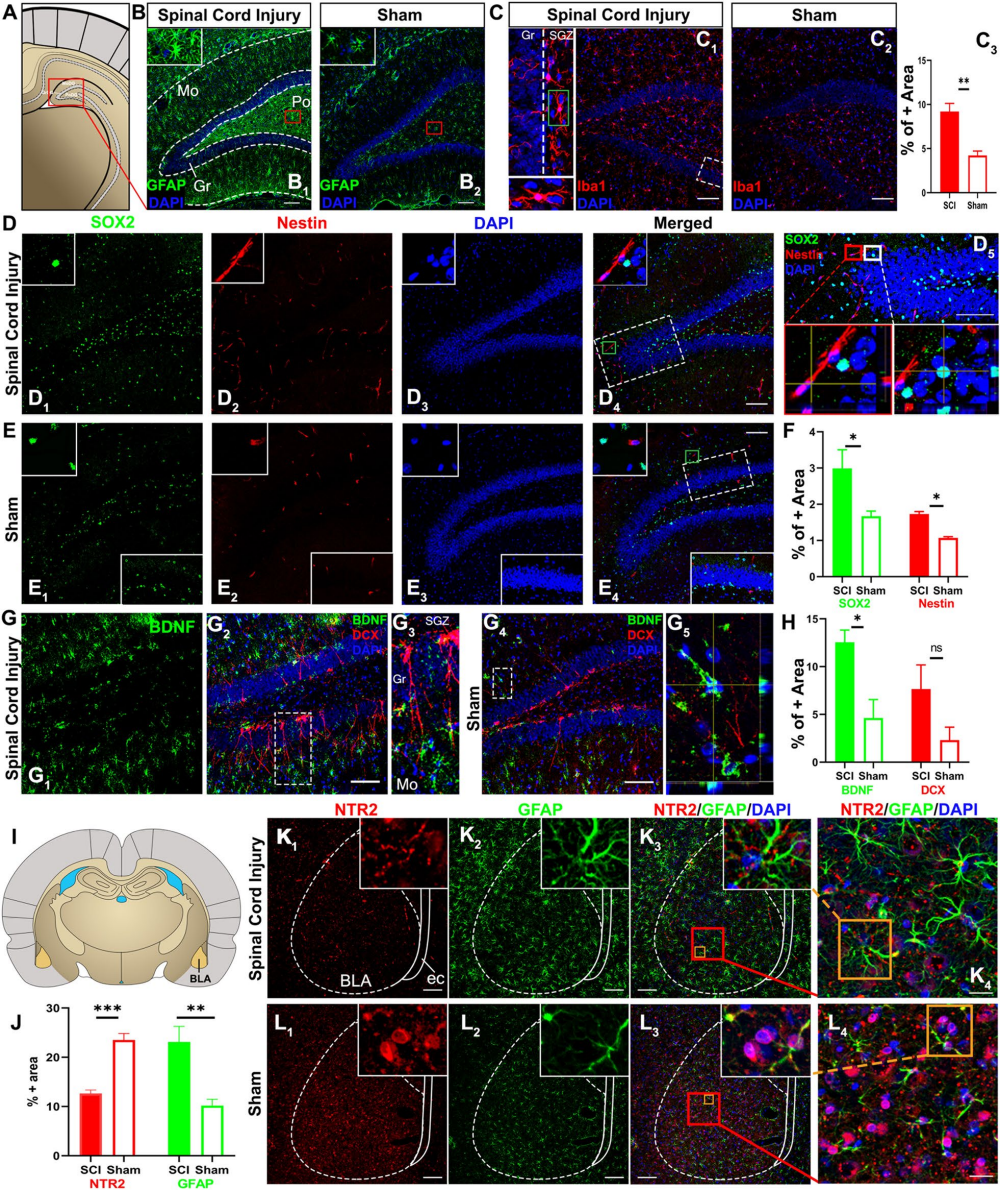
Neuroinflammatory responses, changes of NSC activity in HPC, and reduction of NTR2 expression in BLA after SCI. The DG (in red rectangle) of the hippocampal formation (Bregma − 4.80 mm; **A**) of a SCI rat showed markedly augmented reactive astrogliosis assessed by GFAP IHC stain (**B**_**1**_ and inset), relative to a control image (**B**_**2**_ and inset). Moreover, the mean IRL of Iba1 (red) expressed by hypertrophic microglia (**C**_**1**_ inset: lower left column) concentrated mostly in the neurogenic SGZ of the DG in SCI rats (**C**_1_ inset of upper left column: Iba1 images in SGZ versus those in Gr) was higher than the controls (**C**_**2**_); the difference was statistically significant (**C**_**3**_; **P < 0.01; Student’s *t* test; *n* = 4/group). SCI DG sections exhibited much more nuclear SOX2 (green, **D**_**1**_) and cytosol nestin immunostains (red, **D**_**2**_; DAPI in **D**_**3**_) compared to those of control tissue (**E**_**1**–**3**_). The merged z-stack images (**D**_**4**_) showed that some NSCs had SOX2 (green) and DAPI (blue) co-stained nuclei (cyan in **D**_**5**_). The differences between the IRL means of the two groups were significant (**F**; *P < 0.05; Mann–Whitney *U* test; *n* = 4/group for both SOX2 and nestin). The SCI HPC sections (**G**_**1–3**_), relative to controls (**G**_**4**_), exhibited significantly stronger BDNF immunosignal (green, **G**_**1**_) in cells that were mostly negative for DCX (red, **G**_**2**_). Some DCX+ NSCs also expressed BDNF (yellow cells in **G**_**3**_). The mean IRLs of BDNF, not DCX, were significantly different between the two groups (*P < 0.05 or ns, Mann–Whitney *U* test, respectively; *n* = 4/group; **H**). SCI-triggered neuroinflammatory impacts were evaluated in the BLA (Bregma − 2.30 mm; **I**). Mean IRL of NTR2 (red) or GFAP (green) in the SCI group (**K**_**1**–**3**_) was significantly lower or higher than the control group (**L**_**1**_–**L**_**3**_), respectively (**J**; ***P < 0.001 for NTR2; **P < 0.01 for GFAP; Student’s *t* test; *n* = 4/group). The NTR2 immunostain was mainly located in the cytosol fractions of GFAP− cells, suggesting the cells were BLA interneurons (see morphologic differences between NTR2+ cells/**L**_**4**_ and GFAP+ astrocytes/**K**_**4**_). Scale bars:100 µm except for **K**_**4**_ and **L**_**4**_ where they were 20 µm. *BLA* basolateral amygdala, *DCX* doublecortin, *DG* dentate gyrus, *ec* external capsule, *Gr* granular layer of DG, *HPC* hippocampus, *Mo* molecular layer of DG, *ns* not significant, *NSC* neural stem cell, *NTR2* neurotensin receptor type 2, *Po* polymorphic layer of DG (i.e., hilus), *SGZ* subgranular zone of DG

The subcortical structures primarily consist of the limbic system (i.e., the hypothalamus, the amygdala, the thalamus, and the hippocampus), basal ganglia, and olfactory bulb [54, 55], which play roles in chronic pain-induced depression, anxiety, perception, cognition, and memory [55–58]. Our investigation of the hippocampal formation (Fig. 8A) revealed that the dentate gyrus (DG) cortex of the SCI animals had a qualitatively higher level of astrocytic GFAP (Fig. 8B_1_) compared to laminectomy controls (Fig. 8B_2_). Due to the definitive difference in GFAP IRL observed (i.e., cytoarchitecture of Fig. 8B_1_ inset versus that of Fig. 8B_2_ inset), no statistical comparison was made. Also, there were more areas occupied by Iba-1 expressing activated microglia, mostly in the neurogenic subgranular zone (SGZ; Fig. 8C_1_ and inset), in the DG of the SCI animals than the controls (Fig. 8C_2_); the difference was statistically significant (Fig. 8C_3_; *p* < 0.01; Student’s *t* test; *n* = 4/group).

Importantly, the SCI DG sections were more compactly settled by nuclear Sox2+ (Fig. 8D_1_) and cytoplasm nestin+ NSCs (Fig. 8D_2_; DAPI nuclear counterstain in Fig. 8D_3_; merged image in Fig. 8D_4_), with some early phase NSCs expressing nuclear Sox2 only (see confocal orthogonal slices in Fig. 8D_5_). Conversely, fewer NSCs presented in the control section (Fig. 8E_1–4_), rendering the mean IRL of Sox2 and nestin significantly lower than the SCI group (Fig. 8F; *p* < 0.05; Mann–Whitney *U* test; *n* = 4/group). Further, tissues of SCI animals (Fig. 8G_1–3_), relative to controls (Fig. 8G_4_), had significantly elevated expression of BDNF (Fig. 8G_1_) in cells that were mostly negative for doublecortin (DCX), a marker for migrating NSCs (Fig. 8G_2–5_). Most BDNF+ cells showed features of activated astrocyte or microglia (comparing Fig. 8G_2_ to Fig. 8B_1_ and C_2_); however, some DCX+ NSCs also produced BDNF (i.e., co-stained cells in Fig. 8G_3_: optical slices in Fig. 8G_5_). The mean IRL of BDNF, not DCX, was significantly different between the two groups (Fig. 8H, *p* < 0.05, Mann–Whitney *U* test; *n* = 4/group).

The BLA (Fig. 8I) is an important member of the fear extinction circuitry [59]. Neurotensin receptor 2 (NTR2) expressing neurons in the BLA were recently found to play a crucial role in fear inhibition [60]. The BLA tissue of the SCI group had an average IRL of NTR2 or GFAP that was significantly lower or higher, respectively, than the control group (*p* < 0.001 and 0.01, respectively; Student’s *t* test; *n* = 4/group; Fig. 8J). The BLA of SCI animals (Fig. 8K), compared to controls (Fig. 8L), expressed discernibly less NTR2 (Fig. 8K_1_ versus L_1_; merged image: Fig. 8K_3_ versus L_3_) and had reduced IHC intensity of NTR2 in cell bodies (Fig. 8K_4_ versus L_4_; inset: Fig. 8K_1_ versus L_1_). Conversely, IRL of GFAP was markedly increased (Fig. 8K_2_ versus L_2_; merged image: Fig. 8K_3_ versus L_3_) in hypertrophic astrocytes (inset: Fig. 8K_2_ versus L_2_; higher magnification image: Fig. 8K_4_ versus L_4_) in the SCI group relative to the control group. Noticeably, IHC morphological profiles showed that NTR2 was mostly expressed in neuronal bodies and neurites (i.e., GFAP− cells); however, some GFAP+ astrocytes also expressed NTR2 (i.e., yellow cells in Fig. 8K_3_, L_3_ insets) as reported previously [61].

## Discussion

Chronic pain is one of the most frequent and debilitating complications of SCI [62–65]. NP, defined by the International Association for the Study of Pain (updated Dec. 14, 2017) as “pain caused by a lesion or disease of the somatosensory nervous system”, affects more than half of people who have experienced SCI [2, 3]. Conventional therapeutics such as lithium, gabapentin, and tramadol, which are designed to tackle a single target of neurotransmission, often exhibit short term efficacy with considerable side effects [66–69]. To date, reliable management of clinical SCI pain, especially, NP, remains an unmet medical demand [62, 66]. The situation is partly attributable to a lack of a comprehensive understanding of the systemic scale, dimension, and diversity of pathophysiological mechanisms of NP [2, 62, 66].

Clinical NP manifests as spontaneous pain and/or evoked pain (with less frequency); it occurs as modality-specific sensory gain or loss (e.g., allodynia, hyperpathia and hyperalgesia, or anesthesia dolorosa). Thus, NP is difficult to define in experimental settings, and therapeutics that are effective in laboratory studies often do not translate directly to patients [23]. Animal models of evoked limb withdrawal are commonly used to detect allodynia and hyperalgesia-like hypersensitivities, but they may reflect only the evoked pain component in patients [70]. Moreover, there still lacks a standard model to measure “spontaneous pain” despite using the conditioned place preference (CPP) assay, as CPP typically detects the rewarding and aversive (i.e., reinforcing) effects of drugs. The assessment of spontaneous pain in rodents has become a multiplex subject of extensive debate [71].

In theory, there are multiple potential biomarkers in pain models that may enable improving clinical relevance predication [72]. In this study, the application of mVF, hot plate, and spinal reflex tests is based on the principle of aligning sensory measurements for animal models with current methods of clinical sensory assessment [73]. On this basis, we propose that the evoked mechanical and thermal responses plus the sensorimotor, histopathological, NIF, NML and NPL parameters for lab models of pain or SCI can be redeployed as sensory profiling tools to generate a multidimensional biomarker profile of NP (MBPN) after neurotrauma; the integration of spontaneous pain appraisal via quantifying pain-related physical/behavioral signs (e.g., porphyrin staining, vocalization, etc.) may further improve clinical relevance of this formula [74]. Our MBPN-based novel approach, which does not merely measure “pain” after experimental SCI, may improve translational strength of the data for future research of NP and analgesic development [24, 75].

NP occurs from a few days to several months after clinical SCI, during which one of the secondary injury processes is NIF, hallmarked by reactive gliosis, microglia/macrophage activation, and heightened production of proinflammatory cytokines from residential and invading inflammatory cells [76–78]. These events, acting alone or in consortium, have been identified as promoters of NP [15, 79–81]. Here, we investigated whether a long-term coexistence of NP and multilocus NIF might present in a rat model of T10 compression (Fig. 1). This SCI model was previously established in our laboratory to emulate clinical quasi-static insults to the spinal cord (e.g., injury resulting from vertebral fracture, abscess, herniated intervertebral disk, arthropathy, or tumor) [26, 82]. The T10 SCI model produced above-, at-, and below-injury level mechanical hypersensitivity in all SCI rats (Fig. 2), in contrast to clinical SCIs that affect about 50% of individuals and show above-level allodynia in the least prevalence [83, 84].

Whereas our finding is consistent with laboratory data published by others [85–87], the field does not appear to have unified mechanistic explanations for these discrepancies. Conceivably, compared to humans, rodents could have much stronger sensitivity and response (including those of NIF/NTM/NML/NPL) to nociceptive experiences, which as a pivotal survival mechanism may underlie their higher rate of NP after SCI. Further, discernibly more wide dynamic range (WDR) neurons were found in DHs 2–3 segments above injury site in the allodynic rats [85], suggesting that rodents, relative to humans, may have a higher amplitude of maladaptive neuroplasticity in DH sensory neurons. The higher prevalence of above-level mechanical allodynia in SCI rats could be attributable to this increased proportion of WDR neurons. Importantly, it has been reported that p.i. above-level allodynia in humans may not be due to the SCI itself, but rather linked with concurrent peripheral nerve injury (PNI) [83, 84], and in SCI models, this could be triggered by peripheral nerve sensitization in response to the secondary injury process [88, 89].

As observed before in the same and other SCI models, NIF at the non-treated epicenter and the lumbar and cervical spinal cord stayed active during the subacute and early chronic phases p.i., with most microglia exhibiting a pro-inflammatory phenotype to worsen p.i. destructive sequelae [14, 26, 76–78]. Accordingly, compared to the laminectomy operation, T10 compression, besides causing sensorimotor and histopathological damages (Figs. 2 and 3), markedly elevated inflammatory responses in (1) RL-I and II of the DH where the peripheral nociceptive signals from dorsal root ganglion (DRG) neurons were transmitted to the secondary sensory neurons (note: for our focus on the CNS, DRGs were not analyzed); (2) the lateral and ventral funiculi that housed the spinothalamic pathway; (3) the DC containing the touch and kinesthesia primary pathways that synapsed in the GrN and cuneate nucleus, and (4) the spinoparabrachial pathway in the lateral and dorsal columns that relays in the PBN, involved in the affective component of pain (Fig. 4) [90]. In these sensory nuclei, chronic NIF characterized by microglial and astrocytic activation plus augmented expression of pro-inflammatory cytokines and iNOS (iNOS+ glia are proinflammatory) coexisted with behavioral abnormalities of sensorimotor reflex, evoked mechanical and thermal hypersensitivity (Figs. 4, 5, 6, 7 and 8), and signs suggestive of spontaneous pain in the SCI group only.

Also assessed was the IHC level of CGRP, a key neurotransmitter released from the C and Aδ afferent fiber terminals for pain and temperature sensation onto the DH substantia gelatinosa neurons [44, 45] and p75NTR, a main modulator of DRG and DH RL-I and II neurons for nociceptive transmission [46]. The significantly increased expression of the two molecules in the DRG neuronal afferent terminals and DH neurons of the SCI rats, relative to the controls, indicated that these neurons were hypersensitive, which might have partly ignited and upheld the NP-like behaviors and signs. This conclusion was supported by the statistical linear regression data that demonstrated significant reverse correlations between IRL of CGRP and p75NTR, and thresholds of the bilateral hot plate test (Fig. 5).

We earlier reported that sensorimotor abnormalities in the same T10 SCI model were associated with perturbated expressions of Homer-1a (an immediate-early gene that is increased by synaptic activity) and 5HT in the distal spinal cord [14, 91]. Changes of these two factors have been linked to pain-induced NPL and sensorimotor NML, respectively [14, 92]. The group average IRLs of Homer-1a and 5HT were significantly higher in the SCI DH than the control DH. Moreover, the IRLs of Homer 1a and 5HT were in significant negative correlation with the bilateral hot plate test thresholds of SCI rats (Fig. 5). The results collectively suggested that the DH neural network under this scale of hypersensitivity was still in a state attempting to modulate the elevated nociceptive input via enhancement of Homer-1a and 5HT expression. Since treating rats 3 weeks after the same type of SCI with huperzine A, a multimodal neuromodulation drug, markedly impeded NIF, DH expression of Homer-1a, and NP-like behaviors [14], future therapeutic development should target these neurodynamic events to manage SCI NP [50, 91–93].

Lumbar NIF responses, such as activation of astrocytes and microglia in the lumbar DH after T13 hemisection [93] or L5 microglia activation/↑TNFα and IL1β post-midthoracic SCI [94], have been associated with below-injury level allodynia; in addition, supraspinal NIF and NPL reactions were found in animals with SCI NP [94–96]. We thereby decided to systematically examine possible presence of NIF in major sensory relay and modulation centers concerning allodynia, nocifensive, and affective processes in the brainstem and brain subcortical structures. In the brainstem, GrN neuronal sensitization has been correlated with development of NP and tactile allodynia in the lower limbs [97, 98]. Here, IRL of the immediate-early gene cFos, a marker of pain-related DH neuronal activation [14, 99], was significantly higher in the GrN neurons (Fig. 6) in the context of activated microglia and astrocytes residing in the same region. The changes were coupled with NP-like behaviors of the SCI rats 8 weeks after SCI. Located farther rostral in the brainstem are LPBN neurons that are involved in the affective component of pain by transmitting visceral and somatic sensory information to the forebrain [51]. Published data suggested that LPBN CGRP+ neurons could transmit nociceptive signals via innervation of the paraventricular thalamic nucleus and central amygdaloid nucleus [51, 52]. Rats with chronic hyperalgesia post-T10 compression showed elevated group average scales of astrocytic and microglial activation, IRL of proinflammatory cytokines, and numbers of cFos+/CGRP+ neurons in the LPBN (Fig. 7), reconfirming a possible interaction between PBN NIF, LPBN CGRP+ neuronal hyperactivity, and NP in the chronic phase of SCI.

The descending pain modulatory system (DPMS) circuits consist of the anterior cingulate cortex, amygdala, anterior insula, middle frontal gyrus, RVM, and PAG [100]. The NRM, residing inside the RVM of the brainstem with input from PAG, is the principal source of CNS serotonergic projections including those to the DH, to biphasically modulate nociceptive control during adulthood [101]. However, there has essentially been no published data about possible connections between NRM NIF, altered 5HT expression in NRM or DH, and NP after SCI. In our investigation, significantly higher group mean GFAP IRL was found in hypertrophic astrocytes within NRM of SCI rats only (Fig. 6). Conversely, post-SCI NRM 5HT IRL was markedly reduced (Fig. 6), which was likely caused by chronic NIF [102], as well as surged supply demand of 5HT from RL-I and II of DH (Fig. 5) due to ongoing SCI NP [101]. Correspondingly, the VLPAG of SCI animals had significantly higher IRL of NIF markers (i.e., ↑GFAP, ↑Iba-1, and ↑TNFα) and expression of GAD-67 (Fig. 7), which metabolizes glutamate into GABA (an inhibitory neurotransmitter) and is a marker of GABAergic neurons. As a consequence, the reduced capacity of NRM serotonin and raised PAG GABA in chronic SCI could attenuate descending 5HT-based pain modulation and endogenous opioid-mediated analgesic effect, respectively, as well as conjointly disrupt the PAG–NRM–DH pain suppression pathway in DPMS to promote NP [100, 103, 104].

A feedforward inhibition enabled by BLA via a prefrontal cortex–PAG–NRM–spinal cord pathway for descending noradrenergic and serotoninergic modulation of afferent pain signals, plays a pivotal role in preventing mechanical and thermal hypersensitivity [105]. Furthermore, the spinoparabrachial pathway projects to BLA via PBN for sensory functions [106], and most BLA neurons innervate the hippocampus and neocortex to form bidirectional communications involved in emotion-related memory concerning depression [107, 108]. Indeed, a population of fear-off neurons expressing NTR2 has been identified in BLA for anxiety regulation [60, 109], and neurotensin and NTR2 were shown to directly modulate pain resulting from inflammatory stimuli [110]. Our examination of BLA revealed that SCI, relative to laminectomy alone, significantly heightened reactive gliosis (↑GFAP) but lowered neuronal IRL of NTR2 in the BLA (Fig. 8). The data suggested that T10 compression might interfere with BLA function by inducing NIF and weakening the function of fear-off neurons, which could contribute to development of NP and pain-triggered mood disorders [111].

For post-PNI chronic pain-evoked cognitive deficit, memory dysfunction, anxiety, and depression, NIF and neurogenic changes in the hippocampus, the key structure to process memory, learning, and emotion, have been identified as primary operators [112–115]. Yet, no published data has been available concerning associations between lower thoracic SCI, NP, and alterations of hippocampal NIF and neurogenesis. In this study, T10 compression, relative to laminectomy, significantly increased NIF and NSC presence in the hippocampus of rats with chronic SCI NP (Fig. 8). However, the two groups had comparable IRLs of hippocampal DCX, a marker of migratory NSCs, indicating that SCI caused neurogenic abnormalities beyond NSC proliferation (e.g., ↓migration of newly generated NSCs). This together with the heightened expression of BDNF, which appeared to be produced by the activated glia and NSCs (Fig. 8), could trigger hippocampal synaptic disorders as seen in a PNI model of NP to underlie pain-induced cognitive, mood, and memory problems [114, 116]. In fact, down-regulation of adult hippocampal NSC proliferation reversibly blocked persistent pain, whereas increased NSC activity lengthened pain behaviors in murine models of PNI NP and hindpaw inflammatory pain [115].

## Conclusions

**Fig. 9:**
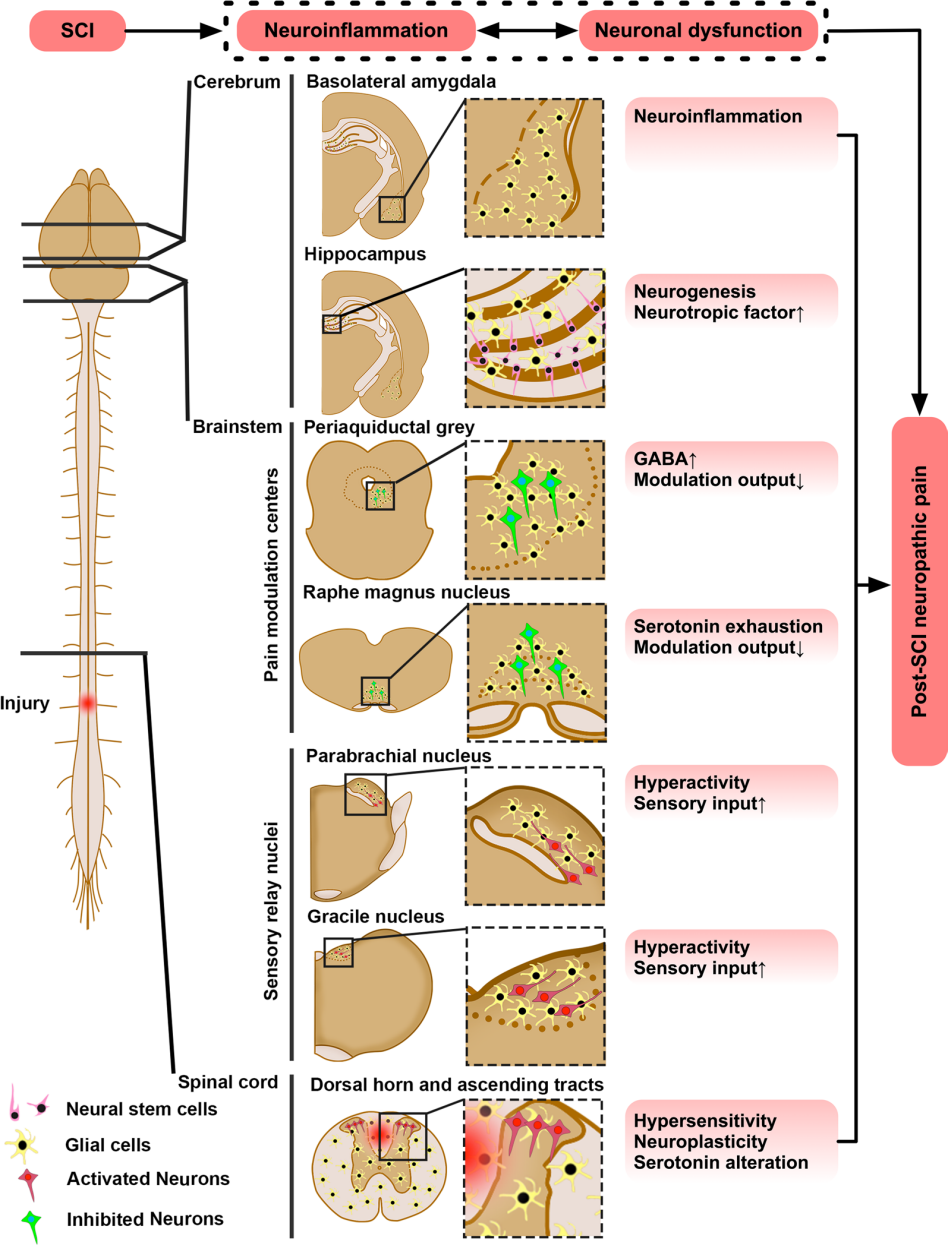
Summary diagram. The data of this study (see details in Figs. 1, 2, 3, 4, 5, 6, 7 and 8) suggested that T10 compression in young adult female rats resulted in coexistence of chronic neuropathic pain (NP) and multilevel neuroinflammation, neurotransmission, neuroplasticity, and neuromodulation responses along the neuroaxis. Such injury-induced neural pathophysiological events in main spinal cord and brain sensory/pain processing centers have generated novel biomarkers as potential mechanistic underpinnings of SCI NP. The information can be used to multidimensionally profile experimental NP to enhance clinical relevance of pain modeling for therapeutic development

To our best knowledge, this is the first study where NP-like behaviors and multilevel NIF, NML, and NPL changes in the CNS have been comprehensively characterized following a focal lower thoracic spinal cord trauma (Fig. 9). Limitations of this work included a single timepoint evaluation of NIF at 8 weeks p.i., no pharmacological or genetic manipulation to determine any causal relationship between hyperalgesia and specific biomarkers, no use of video-based analysis of persistent pain (e.g., the Grimace Scale) [117], and no inclusion of male animals despite the fact that female rats have conventionally been used in SCI modeling for better post-care results and more efficient development of hypersensitivity [118]. These aspects and the relationship between p.i. NP and possible NIF in the somatosensory cortex (which is beyond the scope of the current study) should be investigated in the future. Overall, the data have provided systematic biomarkers as mechanistic leads to profile SCI NP. We concluded that this model of SCI and its outcome measures have established a pertinent regimen to multidimensionally investigate p.i. NP, which may facilitate identification and translation of novel therapeutic targets for devising multimodal treatments of NP after SCI.

## Supplementary Information

**Additional file 1: Table S1.** Scores of spontaneous pain-related physical parameters.

**Additional file 2: Table S2.** Immunohistochemical reaction protocols.

**Additional file 3: Table S3.** Antigen retrieval protocol.

## Data Availability

All data will be accessible at written request after the scientific results are published. All experimental materials are commercially available.
