# Supplementary material for: Coexistence of chronic hyperalgesia and multilevel neuroinflammatory responses after experimental SCI: a systematic approach to profiling neuropathic pain
Source: J Neuroinflammation. 2022 Oct 29;19:264. doi: 10.1186/s12974-022-02628-2 (PMC9617391; doi:10.1186/s12974-022-02628-2)
Supplement: Supplementary file 1 — Additional file 1: Table S1. Scores of spontaneous pain-related physical parameters. [file 12974_2022_2628_MOESM1_ESM.docx]

**Table S1.** Scores of Spontaneous Plain-related Physical Parameters

| **Parameters** | **Score** |
| --- | --- |
| Head fur faulty/fur loss (↑grooming) | 1 |
| Head/neck fur lusterless and disheveled (↓grooming) | 5 |
| Narrowed eyes | 1 |
| Porphyrin stains around the eyes | 5 |
| Reduced locomotion inside the housing cage | 1 |
| Frequent squeaking when not being touched | 5 |
| Repeated licking of the same body/limb spot* | 10 (*biting lesion warrants for termination) |
| Vocalization when being gently handled | 10 |
| *Scores suggestive for spontaneous pian* | *≥ 5 (total score points: 38)* |

*References*

1. Carstens E, Moberg GP: Recognizing pain and distress in laboratory animals. *ILAR J* 2000, 41:62-71.
2. Gillingham MB, Clark MD, Dahly EM, Krugner-Higby LA, Ney DM: A comparison of two opioid analgesics for relief of visceral pain induced by intestinal resection in rats. *Contemp Top Lab Anim Sci* 2001, 40:21-26.
3. Kanzler S, Rix A, Czigany Z, Tanaka H, Fukushima K, Kögel B, Pawlowsky K, Tolba RH: Recommendation for severity assessment following liver resection and liver transplantation in rats: Part I. *Lab Anim* 2016, 50:459-467.
