# Supplementary material for: Coexistence of chronic hyperalgesia and multilevel neuroinflammatory responses after experimental SCI: a systematic approach to profiling neuropathic pain
Source: J Neuroinflammation. 2022 Oct 29;19:264. doi: 10.1186/s12974-022-02628-2 (PMC9617391; doi:10.1186/s12974-022-02628-2)
Supplement: Supplementary file 2 — Additional file 2: Table S2. Immunohistochemical reaction protocols. [file 12974_2022_2628_MOESM2_ESM.docx]

**Table S2.** Immunohistochemical Reaction Protocols

- Materials and chemicals

| Materials and chemicals | Provider | Address |
| --- | --- | --- |
| Normal donkey serum (NDS) | Jackson ImmunoResearch | West Grove, PA |
| Phosphate-Buffered Saline (10X), PBS | Boston Bioproduct | Milford, MA |
| Triton X-100 | Bio-Rad | Hercules, CA |
| Antigen Unmasking Solution (100X), Citrate-Based (Cat. #: H-3300-250; containing 1M citrate) | Vector Labs | Burlingame, CA |
| PAP pen | ABCam | Cambridge, MA |

- Antibody selection

Please see **Table 1** for information about all antibodies used in this study

- Solution preparation

| Solutions | Preparation |
| --- | --- |
| 1X PBS (pH: 7.4) | Dilute 100ml 10X PBS into 900ml dH_2_O (distilled, deionized water) |
| Antibody dilution buffer | 1% NDS+0.3%Triton+1X PBS (pH: 7.4) |
| Washing buffer | 0.03%Triton+1X PBS (pH: 7.4) |
| Blocking buffer | 4% NDS+0.3%Triton+1X PBS (pH: 7.4) |

- Immunohistochemistry (IHC) protocol:

1. Take out the frozen slides from the -80 °C or -20 °C freezer.
2. Thoroughly dry the slides for 2 hours in room temperature (RT).
3. According to the protocol of the laboratory, dilute primary antibodies into specific concentration in dilution buffer (see **Table 1** for details).
4. Using a PAP pen to circle each sample on the slide.
5. Rehydrate the tissue sections on slides in Coplin^®^ glass jars in 1X PBS for 5 mins at RT (note: see **Table S2** regarding the antigen retrieval procedures that were started following Step-5 for the IHC staining of certain molecular markers).
6. Dry the back and areas surrounding each tissue section on the front of the slides using Kimtech^®^ 34155 Kimwipes Delicate Task Wipers.
7. Add blocking buffer solution under RT for about one hour.
8. Use vacuum aspiration to remove the blocking buffer (note: do *not* rinse).
9. Add the primary antibodies in proper dilution ratios listed in **Table 1** (50 µl for each brain section, 25 µl for each spinal cord section). Incubate all slides in a pre-prepared humidified chamber overnight at 4 °C. Alternatively, incubate at RT for 3-4 hours, or inside a 37 °C tissue culture incubator for 1~2 hours.
10. Leave the slides in RT for about 30 minutes after taking them out from an overnight, 3-4 hours or 1-2 hours incubation at 4 °C, RT, and 37 °C, respectively.
11. Rinse the slides in the washing buffer, and immerse the slides into a Coplin^®^ jar containing washing buffer for 5-7 min x 3 times.
12. Dilute the fluorophore-conjugated secondary antibody in the dilution buffer (see **Table 1** for details) under dim light. Overlay the secondary antibody solution on the tissue: 50 µl for each brain section, and 25 µl for each spinal cord section).
13. Incubate the slides in a humidified chamber for one hour at room temperature, protecting from light.
14. Rinse the slides in washing buffer, immerse the slide with the tissue into a Coplin^®^ jar containing washing buffer for 7-10 minutes x 3 times.
15. Coverslipping the tissue sections on each slide with VECTASHIELD^®^ Antifade Mounting Medium with DAPI. Remove the excess mounting media with Kimwipes wipers (note: avoiding or eliminating all air bubbles).
16. The slides are ready for microscopic imaging analysis.

*Abbreviations: Coplin: William M. L. Coplin, M.D. who invented the grooved glass jar. DAPI, 4’,6-diamidino-2-phenylindole. dH2O, distilled, deionized water. IHC, Immunohistochemistry. NDS, Normal donkey serum. PAP, Peroxidase-antiperoxidase. PBS, Phosphate-buffered saline. RT, Room temperature.*
