# Supplementary material for: Coexistence of chronic hyperalgesia and multilevel neuroinflammatory responses after experimental SCI: a systematic approach to profiling neuropathic pain
Source: J Neuroinflammation. 2022 Oct 29;19:264. doi: 10.1186/s12974-022-02628-2 (PMC9617391; doi:10.1186/s12974-022-02628-2)
Supplement: Supplementary file 3 — Additional file 3: Table S3. Antigen retrieval protocol. [file 12974_2022_2628_MOESM3_ESM.docx]

**Table S3.** Antigen Retrieval Protocol

- Antigen Retrieval Methods

Antigen retrieval was performed prior to performing the standard immunohistochemical (IHC) procedures for the following molecular markers (i.e., Steps 6-16 in **Table S1**): 1) calcitonin gene-related peptide (CGRP), p75 neurotrophin receptor (p75NTR), serotonin (5HT), and Homer-1a in the spinal sections 5 mm rostral to the lesion epicenter; 2) glial fibrillary acidic protein (GFAP), inducible nitric oxide synthase (iNOS), ionized calcium-binding adapter molecule-1 (Iba-1), tumor necrosis factor-alpha (TNF𝛼), cFos, NeuN and CGRP in gracile and parabrachial nucleus (see **Table 1** for details).

1. After the first 5 steps described in the standard IHC protocol (see **Table S1**), the slides were immersed into the antigen retrieval solution (i.e., Antigen Unmasking Solution [100X], Citrate-Based [H-3300-250; containing 1M citrate], Vector Labs, Burlingame, CA; working solution: 1:100 dilution in dH_2_O [containing 0.01M citrate]) in a staining dish under 85˚C for 15 minutes.
2. Cool the staining dish in room temperature or on ice for 10~15 minutes.
3. Wash the slides with 1X PBS 5 min for three times.
4. Continue the IHC protocol as per Steps 6-16 (**Table S1**).

*Additional references*

1. Hu BY, Liu XJ, Qiang R, Jiang ZL, Xu LH, Wang GH, Li X, Peng B: Treatment with ginseng total saponins improves the neurorestoration of rat after traumatic brain injury. *J Ethnopharmacol* 2014, 155:1243-1255.

2. Yu D, Neeley WL, Pritchard CD, Slotkin JR, Woodard EJ, Langer R, Teng YD: Blockade of peroxynitrite-induced neural stem cell death in the acutely injured spinal cord by drug-releasing polymer. *Stem Cells* 2009, 27:1212-1222.

3. Yu D, Thakor DK, Han IB, Ropper AE, Haragopal H, Sidman RL, Zafonte R, Schachter SC, Teng YD: Alleviation of chronic pain following rat spinal cord compression injury with multimodal actions of huperzine A. *Proceedings of the National Academy of Sciences of the United States of America* 2013, 110:201300083.

4. Jiao Y, Sun Z, Lee T, Fusco FR, Kimble TD, Meade CA, Cuthbertson S, Reiner A: A simple and sensitive antigen retrieval method for free-floating and slide-mounted tissue sections. *J Neurosci Methods* 1999, 93:149-162.

5. Ino H: Antigen retrieval by heating en bloc for pre-fixed frozen material. *J Histochem Cytochem* 2003, 51:995-1003.

6. Guo W, Miyoshi K, Dubner R, Gu M, Li M, Liu J, Yang J, Zou S, Ren K, Noguchi K, Wei F: Spinal 5-HT3 receptors mediate descending facilitation and contribute to behavioral hypersensitivity via a reciprocal neuron-glial signaling cascade. *Mol Pain* 2014, 10:35.

7. Segi-Nishida E, Warner-Schmidt JL, Duman RS: Electroconvulsive seizure and VEGF increase the proliferation of neural stem-like cells in rat hippocampus. *Proc Natl Acad Sci U S A* 2008, 105:11352-11357.

8. Antibody protocol how-to-book. Novus Biologicals. https://images.novusbio.com/design/labbook.pdf

9. User guide for Antigen Unmasking Solution (citrate-based; pH 6.0; Cat. No.: H-3300; Vector Labs, Burlingame, CA). https://vectorlabs.com/media/folio3/productattachments/protocol/H-3300.UserGuide.10771.RevB.IH.pdf

10. Immunohistochemistry (IHC) Handbook - Novus Biologicals

https://images.novusbio.com/design/BR_IHCGuide_011017_web.pdf
